# Constituents and Metabolites of a French Oak Wood Extract (Robuvit^®^) in Serum and Blood Cell Samples of Women Undergoing Hysterectomy

**DOI:** 10.3389/fphar.2020.00074

**Published:** 2020-02-26

**Authors:** Linda Volpp, Vladimír Ferianec, Miriam Ježovičová, Zdeňka Ďuračková, Oliver Scherf-Clavel, Petra Högger

**Affiliations:** ^1^ Institut für Pharmazie und Lebensmittelchemie, Universität Würzburg, Würzburg, Germany; ^2^ 2nd Department of Gynecology and Obstetrics, Faculty of Medicine, Comenius University, Bratislava, Slovakia; ^3^ Faculty of Medicine, Institute of Medical Chemistry, Biochemistry and Clinical Biochemistry, Comenius University, Bratislava, Slovakia

**Keywords:** polyphenols, urolithins, oak wood, post-surgery recovery, human, liquid chromatography electrospray ionization tandem mass spectrometry

## Abstract

Ellagitannins are signature constituents of oak wood and their consumption has been associated with various health benefits. *In vivo*, they undergo metabolic degradation including gut microbial metabolism yielding urolithins. Only limited data is available about compounds being present in blood after intake of an extract from French oak wood, Robuvit^®^. In the course of a randomized, double-blind, controlled clinical investigation, 66 patients undergoing hysterectomy received placebo or 300 mg Robuvit^®^ per day before and over 8 weeks after surgery. Serum and blood cell samples were analyzed by liquid chromatography electrospray ionization tandem mass spectrometry (LC-ESI-MS/MS). The number of urolithin producers and the urolithin levels increased after intake of Robuvit^®^. In serum samples, the median concentration of urolithin A was 14.0 ng/ml [interquartile range (IQR) 57.4] after 8 weeks. Urolithin B was determined at 22.3 ng/ml (IQR 12.6), urolithin C at 2.66 ng/ml (IQR 2.08). In blood cells, lower concentrations and only urolithins A and B were detected. A statistically significant association of lower post-surgical pain scores with metabotype A was detected (p < 0.05). To conclude, supplementation with French oak wood extract raised urolithin generation in patients and suggested health advantages for urolithin-producers.

## Introduction

Polyphenols are a structurally diverse group of secondary plant metabolites, which are regularly ingested by humans *via* vegetables, fruits, and nuts. Numerous studies investigating the impact of polyphenol-rich diets attributed these nutritional components health-promoting effects such as in cardiovascular and metabolic disease prevention as well as anti-inflammatory, anti-tumor, and neuroprotective activities (Koch, 2019).

Widely distributed and important polyphenolic polymers are condensed tannins (proanthocyanidins) and hydrolysable tannins (ellagitannins) (Beecher, 2003). Ellagitannins occur naturally in some fruits (pomegranate, strawberry, blackberry, raspberry), nuts (walnuts, almonds), and seeds (Lipinska et al., 2014). While ellagitannins cannot be absorbed due to their high molecular weight, some of their metabolites have been found in blood samples and tissues. Ellagitannins undergo various metabolic modifications on their passage through the gastrointestinal tract (Garcia-Munoz and Vaillant, 2014). They are hydrolyzed to release ellagic acid (Lipinska et al., 2014), which is converted by the gut microbiota yielding urolithins (Tomas-Barberan et al., 2014). Urolithins have gained tremendous interest since it has been shown that, for example, urolithin A counteracted molecular aging processes and improved exercise capacity (Ryu et al., 2016) and urolithin B has been discussed to prevent loss of muscle mass (Rodriguez et al., 2017).

Ellagitannins are structurally variable and complex (Zhang et al., 2015). Recently, some ellagitannin structures needed revision after re-examination with modern analytical methods (Yamada et al., 2018). Ellagitannin monomers such as vescalagin or castalagin and dimers such as roburins are found in oak wood. They can serve as signature compounds for distinguishing different oak species (Zhang et al., 2015). Since oak wood is used for barrel making, ellagitannins are transferred into wine or spirits during the aging and storing process and contribute to sensory characteristics of the alcoholic beverages (Glabasnia and Hofmann, 2006).

Ellagitannins are also major components in the dietary supplement Robuvit^®^, a registered proprietary extract from French oak (*Quercus robur L.*) wood. Robuvit^®^ contains e.g., gallic acid, ellagic acid, vescalagin, and castalagin. The extract is standardized according to its HPLC profile and its polyphenol content (> 40%). The specific phenolic composition and quantity have been previously analyzed and described in detail (Natella et al., 2014). In pilot studies with human volunteers, Robuvit^®^ increased the serum antioxidant capacity and the activity of key anti-oxidant enzymes (Horvathova et al., 2014) and reduced symptoms of fatigue (Belcaro et al., 2014; Orszaghova et al., 2015). Based on these observations, a randomized, placebo-controlled, double-blind clinical trial investigating the effects of a dietary supplementation with Robuvit^®^ on post-surgery recovery and quality of life was initiated. Briefly, over 8 weeks female patients ingested placebo or 300 mg Robuvit^®^ per day after a scheduled hysterectomy. Various clinical parameters significantly improved in the supplement group compared to placebo controls (Ferianec et al., 2019). The present investigation was piggybacked to that study to address some relevant additional questions.

**Figure 1:**
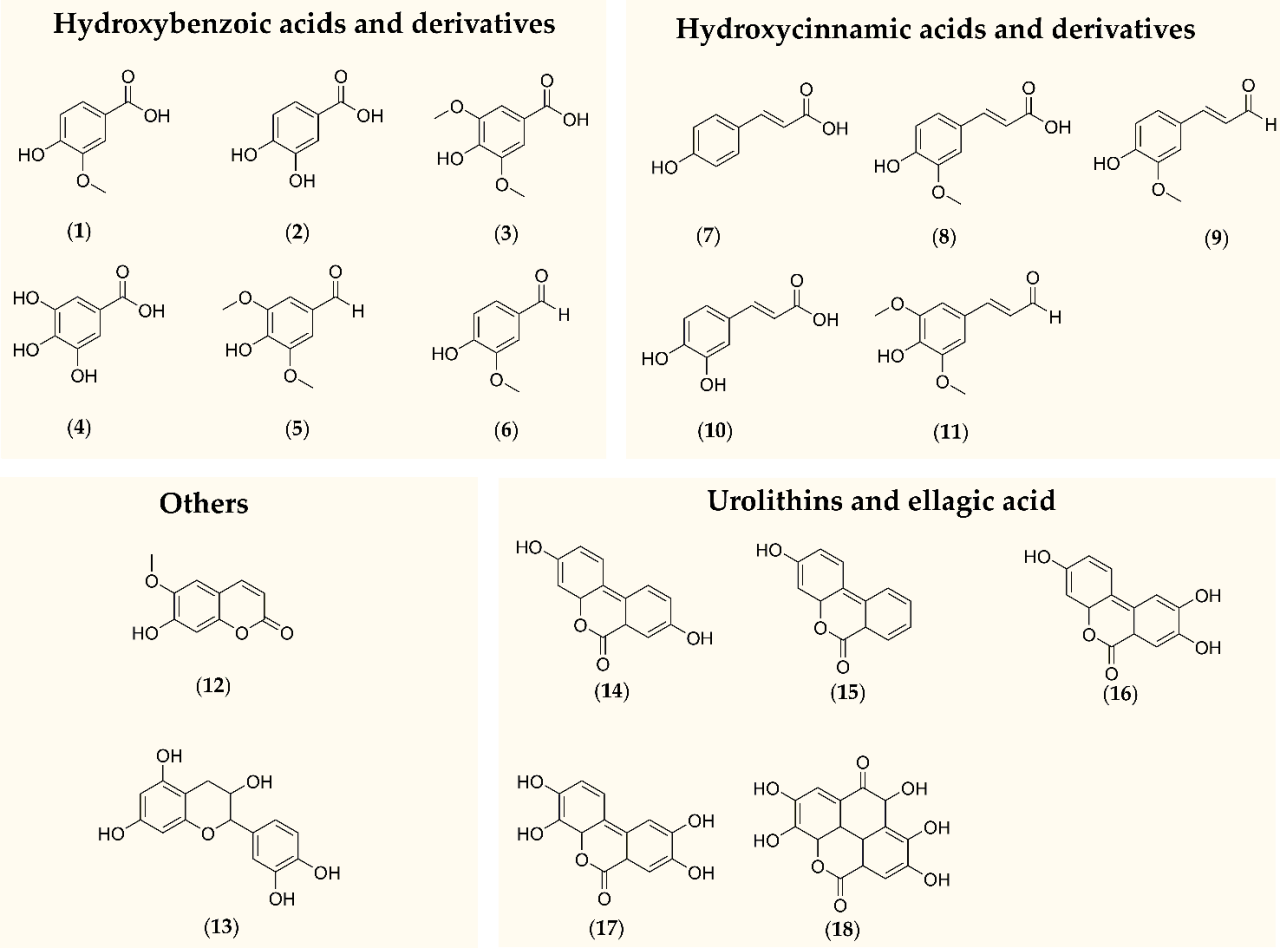
Polyphenols and metabolites that were subject of this study. (1) Vanillic acid, (2) protocatechuic acid, (3) syringic acid, (4) gallic acid, (5) syringaldehyde, (6) vanillin, (7) para-coumaric acid, (8) ferulic acid, (9) coniferaldehyde, (10) caffeic acid, (11) sinapinaldehyde, (12) scopoletin, (13) +-catechin, (14) urolithin A, (15) urolithin B, (16) urolithin C, (17) urolithin D, (18) ellagic acid.

While animal studies investigating the metabolism of oak components have been reported (Azorin-Ortuno et al., 2008; Gonzalez-Barrio et al., 2012), so far only limited data is available about constituents or metabolites found in humans after oral intake of an oak wood extract. In a pilot study with three volunteers, the absorption of Robuvit^®^ constituents and metabolites has been explored (Natella et al., 2014). Besides gallic and ellagic acid, urolithins A, B, and C glucuronides have been detected in plasma samples after supplementation. It is possible that more than these compounds might be present in blood samples after ingestion of Robuvit^®^ since a multitude of low molecular weight polyphenolic constituents have been determined in oak wood (Cadahia et al., 2001; Zhang et al., 2015). Moreover, in a previous investigation involving analysis of polyphenols after ingestion of a pine bark extract, it was observed that some compounds, for instance catechin and taxifolin, were found at higher concentrations in blood cells compared to serum (Mülek and Högger, 2015). Thus, distribution sites other than serum might play a role for individual polyphenols. The purpose of the present study was to analyze constituents and metabolites of Robuvit^®^ in a bigger cohort of participants. For the first time, concentrations in both serum and blood cells were to be investigated. Therefore, an analytical method for the determination of several polyphenolic constituents reported being present in oak wood (Cadahia et al., 2001; da Silva et al., 2009; Zhang et al., 2015) was developed. Compounds were chosen based on their molecular weight and thus their assumed potential for absorption in the gastrointestinal tract. Additionally, the gut microbial metabolites of ellagitannins, urolithins (Espin et al., 2007b; Kawabata et al., 2019), were to be determined (**Figure 1**).

Although a multitude of studies investigating the dietary effects of polyphenols have been published (Espin et al., 2007a), it is not completely clear which compounds or metabolites thereof actually reach the cells *in vivo* (Williamson et al., 2018; Luca et al., 2019) and which health effects might be related to changes on a cellular level. Therefore, another objective of the present study was to uncover potential correlations of analyte presence and clinical parameters.

## Materials and Methods

### Chemicals and Reagents

The analytical standards caffeic acid, (+) catechin, ellagic acid, ferulic acid, gallic acid, trans para-coumaric acid, 3,4-dihydroxybenzoic acid (protocatechuic acid), urolithin A, urolithin B [3-hydroxyl-6H-benzo(c)chromen-6-one], 4-hydroxyl-3-methoxycinnamaldehyde (coniferyl aldehyde), trans-3,5-dimethoxy-4-hydroxycinnamaldehyde (sinapic aldehyde), scopoletin, syringic aldehyde, syringic acid, vanillic acid, vanillin, and the internal standard 1,2,3-^13^C_3_ ferulic acid were purchased from Sigma Aldrich (Taufkirchen, Germany). Urolithin C and urolithin D were obtained from Biozol (Eching, Germany). Methanol (BAKER ANALYZED LC-MS Reagent), water (HiPerSolv CHROMANORM for HPLC MS grade), acetonitrile (Ultra Gradient HPLC Grade), and L(+) ascorbic acid were purchased from VWR (Darmstadt, Germany). Formic acid, ammonium formate, ß glucuronidase from *Helix pomatia* (type HP 2, aqueous solution), ethyl acetate, disodium ethylenediaminetetraacetic acid (EDTA), magnesium sulfate (anhydrous), sodium acetate, and Dulbecco's phosphate buffered saline were all obtained from Sigma Aldrich.

### Standard Solutions

Stock solutions (1 mg/ml) of each analytical standard and the internal standard (IS) 1,2,3-^13^C_3_ ferulic acid were prepared in methanol, aliquoted and kept at −80°C. They were diluted as needed with methanol to yield a working solution containing all analytes. The working solution containing the IS was prepared separately by diluting the stock solution of the IS to yield a concentration of 2 µg/ml in a mixture of methanol and water (1:1; v/v). The IS working solution was aliquoted and stored at −20°C.

### Human Serum and Blood Cells

Human serum and blood cells for the calibration curves and quality control samples were obtained from a blood bank (Bayerisches Rotes Kreuz, München, Germany). Serum originated from six different donors, was pooled, and stored in aliquots at −20°C. Blood cells were obtained by centrifugation (2,500 x g, 4°C, 20 min) of whole blood and discarding of the upper layer. Blood cells of four different donors were pooled, aliquoted, and kept at −20°C.

Human serum and blood cells from 66 hysterectomy patients were provided by Prof. Z. Ďuračková (Faculty of Medicine, Comenius University, Slovakia). Samples were collected in the course of a randomized, double-blind, placebo-controlled clinical study detailed earlier (Ferianec et al., 2019). Briefly, patients who met the inclusion criteria (n = 66) were randomized to receive 100 mg proprietary oak wood extract (Robuvit^®^, batch number Q086X, provided by Horphag Research Ltd., Geneva, Switzerland) three times daily (daily dose 300 mg) or placebo. Patient blood samples were obtained before surgery (t = 0) and four (t = 4) and eight (t = 8) weeks after surgery. At t = 4 and t = 8, samples were drawn approximately 12 h after the last intake of Robuvit^®^ or placebo. Patients had given written informed consent, the study had been approved by the Ethics Committee of the University Hospital and the Faculty of Medicine, Comenius University Bratislava (10.01.2011) and has been performed in accordance with the ethical standards laid down in the 1964 Declaration of Helsinki and its later amendments. The study has been registered with the study ID number ISRCTN11457040.

### Liquid Chromatography

Samples were analyzed by HPLC MS/MS using two different chromatographic methods. The LC System (Agilent 1200 series) comprised a pump model BinPump SL G1312B, a degasser G1379B and an autosampler HiP ALS SL+ G1312B. A Pursuit PFP column (150 mm x 4.6 mm, particle size 3 µm) (method 1) and a Zorbax SB C18 column (100 mm x 2.1 mm, particle size 3 µm) (method 2) were used for the chromatographic separation (Agilent Technologies, Santa Clara, USA). The mobile phase consisted of an ammonium formate buffer (5 mM) containing 0.065% formic acid (pH 3.2) (A) and methanol with 0.2% formic acid (B).

#### Method 1

For the quantification of urolithin A, urolithin B, (+) catechin, syringic acid, syringaldehyde, ferulic acid, caffeic acid, gallic acid, vanillic acid, para-coumaric acid, protocatechuic acid, sinapic aldehyde, scopoletin, coniferyl aldehyde, and vanillin, the solvent flow was 0.5 ml/min and the injection volume 5 µl. The gradient elution started at 20% B (0–1 min) and gradually increased to 60% B (2 min), was held constant until 6 min, increased further to 90% B (8 min), and 95% B (9 min) was kept until 13 min.

#### Method 2

Urolithin C, urolithin D, and ellagic acid were analyzed using a solvent flow of 0.3 ml/min and an injection volume of 2.5 µl. The gradient elution started with the same initial solvent composition as method 1 (20% B), was increased to 80% B (1.2 min), and held constant until 1.8 min. The ratio of solvent B was raised to 95% until 2 min and maintained for 5 min.

### Mass Spectrometry

For analyte detection a Triple Quadrupole LC/MS G6460A, equipped with an electrospray ion (ESI) source with JetStream (Agilent Technologies, Santa Clara, USA), was used. The mode of detection was a dynamic multiple reaction monitoring (dMRM) with a cycle time of 750 ms in a positive and negative ionization mode.

The drying gas temperature and flow of the ESI source were set at 250°C and 12 L/min respectively, sheath gas temperature and flow were 400°C and 12 L/min. The nebulizer was operated at a pressure of 50 psi and the nozzle voltage was set to 500 V in the positive mode and 0 V in negative mode. The capillary voltage was 4,000 V in the positive mode and 2,500 V in the negative mode. The electron multiplier voltage was set to 400 V (positive) and 500 V (negative). For the fragmentation of precursor ions in the collision cell, nitrogen was used as collision gas. The most abundant precursor ions were [M+H]^+^ and [M−H]^−^ in positive and negative ionization mode, respectively, for all analytes. Fragmentor voltage, collision energy, and cell accelerator voltage were optimized individually for each compound or transition by manually injecting standard substances in methanol (5 µg/ml) (**Supplementary Table S1**). Data acquisition and analysis were performed with MassHunter Workstation B 08.02 (Agilent Technologies, Santa Clara, USA).

### Sample Preparation

#### Human Serum

Serum samples were prepared by liquid liquid extraction after enzymatic hydrolysis of metabolic conjugates with β glucuronidase. Briefly, 500 µl of human serum samples were acidified with 4 µl formic acid (pH 5) and incubated with 8 µl aqueous β glucuronidase solution (>100,000 U/ml) from *Helix pomatia* for 30 min at 37°C on a horizontal shaker (120 rpm). The incubation was stopped with ice. Sixteen microliters of formic acid and 50 µl of the IS working solution (2 µg/ml) were added and samples were vortexed. After addition of 1 ml ethylacetate samples were shaken for 5 min on a rotating mixer. Following phase separation by centrifugation for 5 min at 3,000 x g (Centrifuge 5702, Eppendorf, Hamburg, Germany), 600 µl of the upper organic layer were evaporated under nitrogen until dryness and reconstituted with 200 µl of a mixture of methanol and water (1:1; v/v). Before LC MS/MS analysis, the extracted samples were centrifuged at 18,000 x g for 15 min (4°C).

#### Human Blood Cells

Human blood cells were extracted based on a adapted quick, easy, cheap, effective, rugged and safe (QuEChERS) approach as previously described (Mülek and Högger, 2015) with some modifications. Samples were analyzed after prior enzymatic deconjugation with β glucuronidase. Briefly, 1 g human blood cells were mixed with 50 µl of EDTA-solution (100 mM), 100 µl of ascorbic acid solution (10 mg/ml) and diluted with 4 ml phosphate buffered saline. Thereafter, the cells were acidified with 8 µl formic acid. After addition of 16 µl β glucuronidase from *Helix pomatia*, the samples were incubated for 30 min at 37°C under gently shaking. Subsequently, 50 µl of the IS working solution (2 µg/ml) was added, as well as 2.5 ml acetonitrile containing 1% acetic acid. After vortexing, QuEChERS salts (2 g magnesium sulfate and 500 mg sodium acetate) were added to each sample. The samples were thoroughly mixed on a Multivortex (VW-25010 Multi-Tube Vortexer, VWR, Darmstadt, Germany) and centrifuged to facilitate phase separation (4°C; 10 min., 3,500 x g); 850 µl of the upper layer were transferred to a vial containing 10 µl of ascorbic acid solution (10 mg/ml) and evaporated until dryness using a vacuum centrifuge (Concentrator 5301, Eppendorf, Hamburg, Germany). The residues were reconstituted with 100 µl of a mixture of methanol and water (1:1; v/v). Prior to LC MS/MS analysis, the extracted samples were centrifuged at 18,000 x g for 15 min (4°C).

### Quantification of the Study Samples

Calibration curves were prepared from spiked human pooled serum or blood cells and plotted using weighted linear regression (1/conc²). Study samples were extracted and quantified accordingly. If analytes were already present in the blank matrix, the calibration curves of the respective compounds were shifted along the y-axis depending on the response of the zero-samples (containing IS) as described previously (Mülek and Högger, 2015).

### Method Validation

A validation of the method for the quantification of urolithin A, urolithin B, urolithin C, urolithin D, syringic acid, syringaldehyde, ferulic acid, caffeic acid, gallic acid, vanillic acid, para-coumaric acid, protocatechuic acid, sinapic aldehyde, scopoletin, coniferyl aldehyde, and vanillin in serum was performed according to European Medicines Agency [(EMA), 2011] and United States Department of Health and Human Services and Food and Drug Administration (FDA) guidelines (2018). This comprised accuracy, precision, linearity, lower limit of quantification, dilution integrity, matrix effects, and stability in matrix and stock solutions. For analyte quantification from blood cells, the method was validated for urolithin A, urolithin B, urolithin C, urolithin D, syringic acid, ferulic acid, caffeic acid, gallic acid, vanillic acid, para-coumaric acid, and scopoletin. As analyte-free serum or blood cells were not available, selectivity and carry-over were only assessed for those compounds, which were not detectable in blank matrix (gallic acid, sinapic aldehyde, coniferyl aldehyde, ellagic acid, and urolithin D).

### Statistical Analysis

For descriptive statistics of not normally distributed data, the median and interquartile range (IQR) was calculated instead of mean and standard deviation (SD). Statistical analyses were performed using R version 3.6.0 for Windows. Student's t-test was used for comparison of group differences in case of normally distributed data according to Shapiro-Wilk test. Not normally distributed data was analyzed using Mann-Whitney-U tests. A p-value < 0.05 was considered to be statistically significant.

## Results

### Patient Characteristics and Samples

Of the 70 patients recruited for the study (age 52.9 ± 9.4 years) 66 were randomized to receive three times daily either 100 mg of the oak wood extract Robuvit^®^ (n = 33) or placebo (n = 33) after a medically indicated hysterectomy surgery procedure. The detailed flow diagram of the study has been published earlier (Ferianec et al., 2019). Blood samples were obtained before surgery (t = 0) and four (t = 4) and eight (t = 8) weeks after surgery. Blood samples for analysis were available of 33, 28, and 28 patients at t = 0, t = 4, and t = 8, respectively, in the Robuvit^®^ group and of 31, 21, and 19 patients at t = 0, t = 4, and t = 8, respectively, in the placebo group. In the placebo group, blood cells samples of one patient at t = 0 and of one patient at t = 4 were accidently lost (spilled) therefore only samples of 30 (t = 0) and 20 (t = 4) patients were available for analysis.

### Analytical Method

Serum and blood cells were analyzed separately as previously described (Mülek and Högger, 2015) using a LC-ESI-MS/MS method which was optimized and validated for 16 oak wood constituents and metabolites [**Figure 1**: (1)-(12), (14)-(17)]. Validation parameters such as accuracy, precision, linearity, dilution integrity, matrix effects, and stability are documented in the **Supplementary Materials** (**Supplementary Tables S2**–**S7**). Two additional constituents, catechin and ellagic acid [**Figure 1**: (13) and (18)], were monitored qualitatively, but method parameters were not validated.

For serum, the lower limits of quantification (LLoQ), corresponding to the lowest data point of the calibration range, was lowest for syringaldehyde (0.3 ng/ml (1.5 nM); **Supplementary Table S8**) and urolithin B (0.5 ng/ml (2.4 nM)). The method was less sensitive for protocatechuic acid (LLoQ 13 ng/ml (84.36 nM)) and vanillic acid (LLoQ 17 ng/ml (101.1 nM)).

For blood cell samples, the LLoQ were generally higher than for serum (**Supplementary Table S8**). The method was most sensitive for urolithin B (LLoQ 0.6 ng/g) and urolithin A (LLoQ 1 ng/g) and least sensitive for syringic acid (LLoQ 15 ng/g) and caffeic acid (LLoQ 16 ng/g). For both methods, the linear range used for sample analysis was adapted to the expected analyte concentrations.

### Serum Sample Concentrations

**Figure 2:**
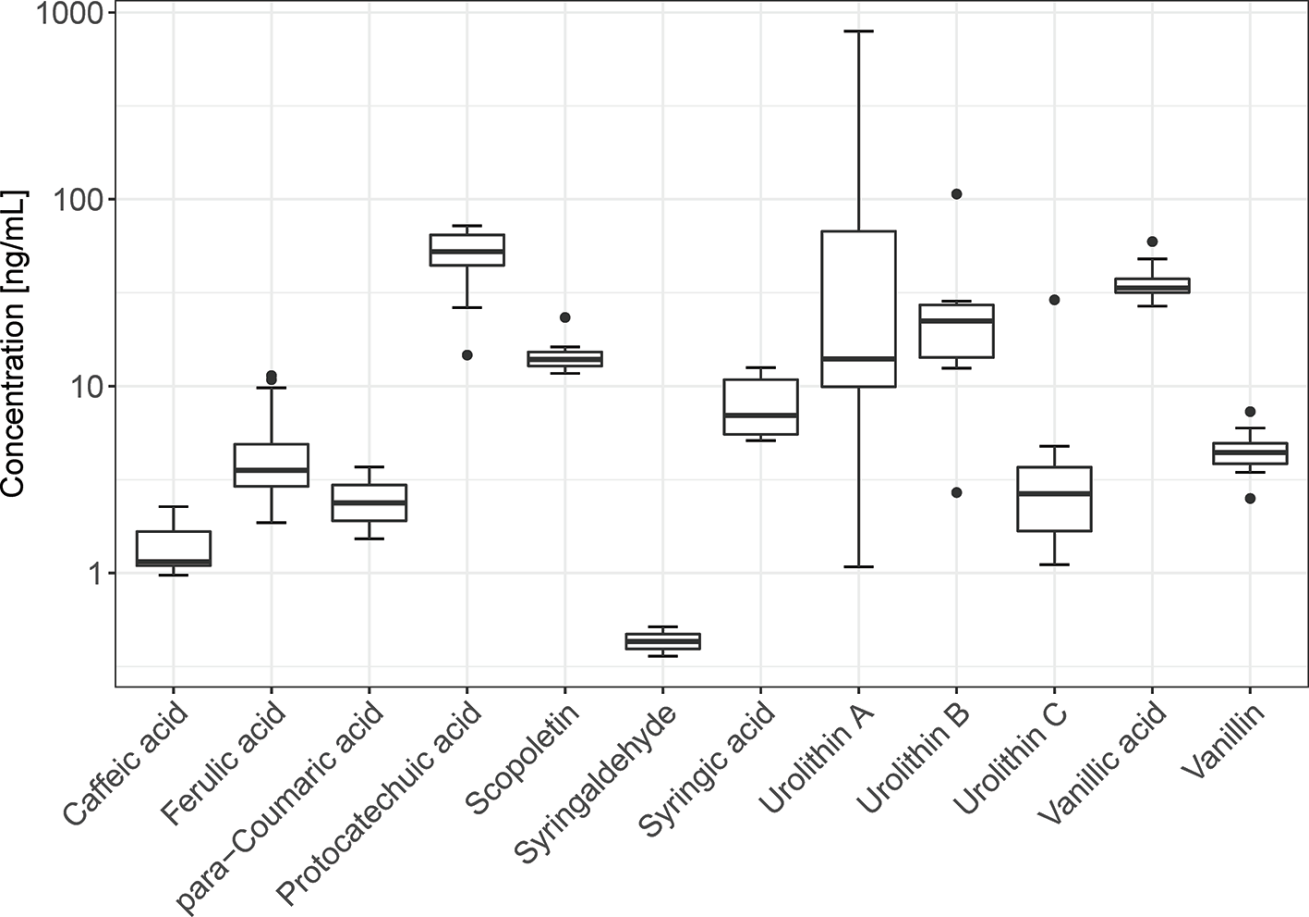
Box-plot of the analytes detected in the patients' serum samples after 8 weeks of daily intake of 300 mg Robuvit^®^. Due to the high inter-individual variability of polyphenol concentrations, the y-axis is in a logarithmical scale.

Of the oak wood constituents and metabolites that were assumed to be detectable in serum samples, 11 compounds were indeed found after 4 weeks (**Table 1**) while 12 compounds were quantified in the patients' samples after 8 weeks of intake of Robuvit^®^ (**Figure 2**). After 4 weeks, p-coumaric acid was not detected yet, but it was present after 8 weeks. Neither after 4, nor after 8 weeks urolithin D, coniferyl aldehyde, sinapin aldehyde, and gallic acid were determined at concentrations above the LLoQ. Neither catechin nor ellagic acid were detected in any patient sample.

**Table 1:** Serum concentrations in the Robuvit^®^ and placebo group after 4 and 8 weeks. All other analytes revealed concentrations below the lower limit of quantification.

Analyte	Robuvit^®^		Placebo	
	t = 4	t = 8	t = 4	t = 8
Caffeic acid	1.22 [0.33]	1.15 [0.57]	1.20 [0.34]	1.11 [0.20]
Ferulic acid	5.10 [2.66]	3.55 [1.99]	5.30 [1.74]	3.82 [3.61]
p-Coumaric acid	< LLoQ	2.61 [1.09]	2.04 [1.01]	1.15 [0.29]
Protocatechuic acid	62.8 [23.6]	52.4 [20.1]	59.8 [11.7]	43.4 [9.83]
Scopoletin	13.2 [2.00]	13.9 [2.42]	13.5 [2.52]	12.2 [3.11]
Syringaldehyde	1.29 [0.72]	0.44 [0.08]	0.74 [−]	< LLoQ
Syringic acid	8.99 [4.01]	6.99 [5.34]	9.27 [3.64]	7.89 [2.98]
Urolithin A	13.2 [31.3]	14.0 [57.4]	18.5 [24.5]	9.13 [8.07]
Urolithin B	20.0 [6.26]	22.3 [12.6]	3.41 [−]	< LLoQ
Urolithin C	2.39 [1.15]	2.66 [2.08]	7.85 [1.44]	0.73 [−]
Vanillic acid	36.6 [6.40]	33.5 [5.79]	37.6 [10.8]	33.2 [7.06]
Vanillin	2.68 [0.73]	4.41 [1.10]	2.80 [0.61]	3.53 [1.11]

Concentration of the analytes in serum (ng/ml) after 4 (t = 4) and 8 (t = 8) weeks ingestion of Robuvit^®^ or placebo. Numbers represent the median with interquartile range (IQR). No IQR is given when only one participant had detectable concentrations. LLoQ, lower limit of quantification.

After 8 weeks intake of Robuvit^®^, highest concentrations were found for protocatechuic acid [median 52.4 ng/ml (340.3 nM); interquartile range (IQR) 20.1 (130.2)], vanillic acid [33.5 ng/ml (199.2 nM); IQR 5.8 (34.4)], scopoletin [13.9 ng/ml (72.5 nM); IQR 2.4 (12.6)], and urolithins A [14.0 ng/ml (61.5 nM); IQR 57.4 (251.6)], and B [22.3 ng/ml (105.3); IQR 12.6 (59.2)]. In the serum of all patients (n = 28; 100%) ferulic acid, protocatechuic acid, scopoletin and vanillic acid were found. In contrast, only two patients had measurable concentrations of p-coumaric acid and syringaldehyde (**Figure 2**).

Various polyphenols were also detectable in serum samples of patients receiving placebo (**Table 1**). After 8 weeks, highest concentrations were found for protocatechuic acid (median 43.4 ng/ml [281.5 nM); IQR 9.8 (63.8)], vanillic acid [33.2 ng/ml (197.5 nM); IQR 7.1 (42.0)], and scopoletin [12.2 ng/ml (63.6 nM); IQR 3.1 (16.2)]. In contrast, urolithins were either not detected (urolithin B) or at low concentrations [urolithin A 9.1 ng/ml (40.0 nM), IQR 8.1 (35.4); urolithin C 0.73 ng/ml in one patient]. In the serum of all patients (n = 19; 100%) protocatechuic acid and scopoletin were found. In contrast, no patient had measurable concentrations of urolithin B and syringaldehyde.

Since no clear differences in most analyte concentrations after intake of either Robuvit^®^ or placebo were seen after 4 and 8 weeks, and since many patients also had basal polyphenol serum concentrations before the intervention (t = 0; **Supplementary Table S9**), only the urolithins were further analyzed in detail. The urolithin concentrations revealed the most pronounced differences between the Robuvit^®^ and the placebo group (**Table 2**). Urolithin A concentrations increased in serum samples after 4 and 8 weeks of Robuvit^®^ intake while this was not consistent for placebo users. Urolithin B was almost exclusively present in serum samples after Robuvit^®^ ingestion and concentrations steeply increased after 4 weeks of use. Compared to 4 weeks, the median concentrations after 8 weeks were not considerably higher. Likewise, in the Robuvit^®^ group, urolithin C levels in serum again increased after 4 and 8 weeks compared to baseline while this was not consistent after placebo intake. Due to the very high inter-individual variability of the data, none of the differences reached statistical significance.

**Table 2:** Concentration of urolithins in serum (ng/ml) of the participating patients before the intervention (t = 0), and after 4 and 8 weeks intake of Robuvit^®^ or placebo (t = 4 and t = 8).

Urolithin	Robuvit^®^			Placebo		
	t = 0	t = 4	t = 8	t = 0	t = 4	t = 8
A	1.80 [0.88]	13.22 [31.28]	14.04 [57.42]	5.80 [12.75]	18.52 [24.51]	9.13 [8.07]
B	1.22 [0.47]	19.97 [6.26]	22.34 [12.56]	< LLoQ	3.41 [−]	< LLoQ
C	1.69 [0.07]	2.39 [1.15]	2.66 [2.08]	0.71 [−]	7.85 [1.44]	0.73 [−]

Numbers represent the median with interquartile range (IQR). No IQR is given when only one participant had detectable concentrations. LLoQ, lower limit of quantification.

All analyte concentrations in serum samples were quantified after hydrolysis of potential sulfate-/glucuronic acid conjugates. To determine whether free urolithins were present, serum samples were also analyzed without prior conjugate hydrolysis. In that case, no measurable concentrations were detected (data not shown) suggesting that the degree of urolithin conjugation was close to 100%.

### Blood Cell Sample Concentrations and Analyte Distribution in Blood

In blood cell samples, only five different polyphenols were determined (**Table 3**). Compared to serum, some analytes were present at higher concentrations in blood cells, namely ferulic acid, scopoletin and vanillic acid. For example, after 8 weeks of intake of Robuvit^®^, ferulic acid was detected at a median concentration of 7.81 ng/g (IQR 2.28), scopoletin at 16.64 ng/g (IQR 3.28), and vanillic acid at 54.58 ng/g (IQR 10.58). In contrast, compared to serum, lower concentrations of urolithins A and B were present in blood cells [median 2.21 ng/g (IQR 15.23) and 2.89 ng/g (IQR 3.04), respectively] and urolithin C was not found at all in blood cells.

**Table 3:** Blood cell concentrations after intake of Robuvit^®^ or placebo.

Robuvit^®^ group			
Analyte	t = 0	t = 4	t = 8
Ferulic acid	7.90 [1.87]	6.34 [0.93]	7.81 [2.28]
Scopoletin	14.30 [3.42]	14.81 [3.32]	16.64 [3.28]
Urolithin A	4.25 [0.61]	2.60 [4.86]	2.21 [15.23]
Urolithin B	17.15 [−]	2.00 [0.41]	2.89 [3.04]
Vanillic acid	51.11 [22.08]	52.27 [9.37]	54.58 [10.58]
**Placebo group**			
**Analyte**	**t = 0**	**t = 4**	**t = 8**
Ferulic acid	7.78 [2.30]	6.49 [1.74]	8.17 [2.43]
Scopoletin	13.41 [3.99]	15.82 [3.37]	15.55 [3.95]
Urolithin A	9.78 [6.17]	2.32 [1.01]	1.52 [4.93]
Urolithin B	<LLoQ	<LLoQ	<LLoQ
Vanillic acid	48.79 [11.90]	56.09 [10.11]	58.57 [15.78]

Concentration of the analytes in blood cells (ng/g blood cells) at baseline (t= 0), and after 4 and 8 weeks (t = 4, t = 8). Numbers represent the median with interquartile range (IQR). No IQR is given when only one participant had detectable concentrations. LLoQ, lower limit of quantification.

Median concentrations of ferulic acid, scopoletin and vanillic acid in blood cells neither clearly varied from baseline to 8 weeks, nor did they clearly differ between the Robuvit^®^ and placebo group (**Table 3**). Unlike in serum samples, the urolithin levels did not increase in blood cells after Robuvit^®^ ingestion. The only unique observation was that urolithin B was consistently not detectable after placebo intake.

If the analytes were present in both serum and blood cells of individual patients, the concentration ratios were calculated. Ferulic acid, scopoletin and vanillic acid preferentially distributed into blood cells with blood cell/serum ratios of 2.17 (IQR 1.41), 1.11 (IQR 0.27) and 1.58 (IQR 0.46), respectively, at t = 8.

**Figure 3:**
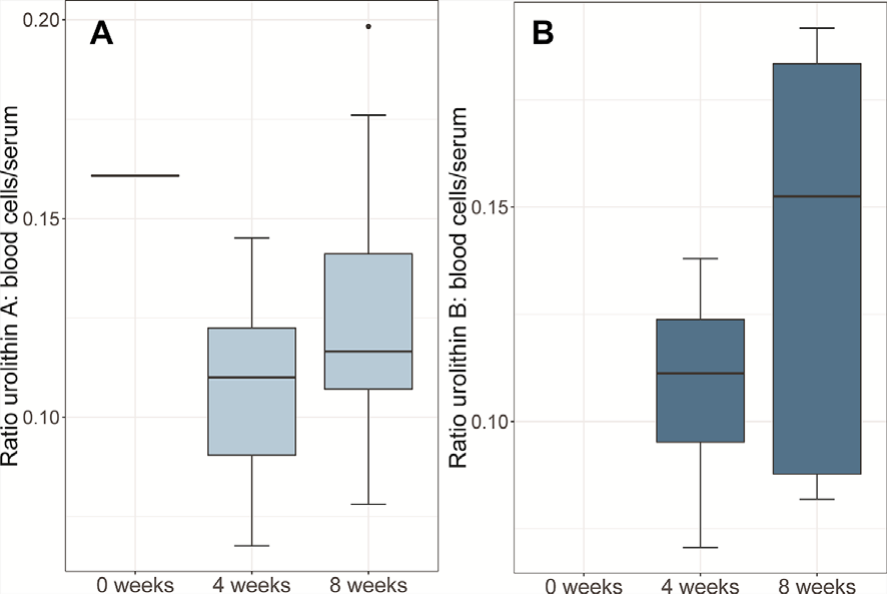
Box-plots of urolithin concentrations ratios in blood cells related to serum after intake of Robuvit^®^ over 8 weeks: **(A)** urolithin A, **(B)** urolithin B. Ratios below 1.0 indicated that the compounds were predominantly present in serum.

For urolithins, all blood cell/serum ratios were clearly below 1.0, indicating that the compounds were primarily present in serum. For urolithin A, the ratio was 0.161 (t = 0; only one patient; **Figure 3A**), 0.110 (t = 4; IQR 0.032) and 0.117 (t = 8; IQR 0.034), indicating that after intake of Robuvit^®^ the compound was not enriched in blood cells. A similar picture was seen for urolithin B with no data for t = 0 and ratios of 0.111 (IQR 0.029) and 0.153 (IQR 0.096) after 4 and 8 weeks, respectively (**Figure 3B**).

### Urolithin Production and Metabotypes

Particularly the urolithins were not detectable in serum or blood cell samples of all patients. After 8 weeks of Robuvit^®^ intake, 75% of the participants produced at least one of the urolithins, 75% produced urolithin A, 21% also urolithin B, and 25% urolithin C. Urolithin D was not detected in any study sample. According to the classification suggested by Tomas-Barberan et al. (2014) the participants were assigned to different phenotypes with “metabotype A” producing urolithin A and maybe urolithin C, “metabotype B” urolithin A and urolithin B and potentially urolithin C, and “metabotype 0” producing no urolithins.

**Figure 4:**
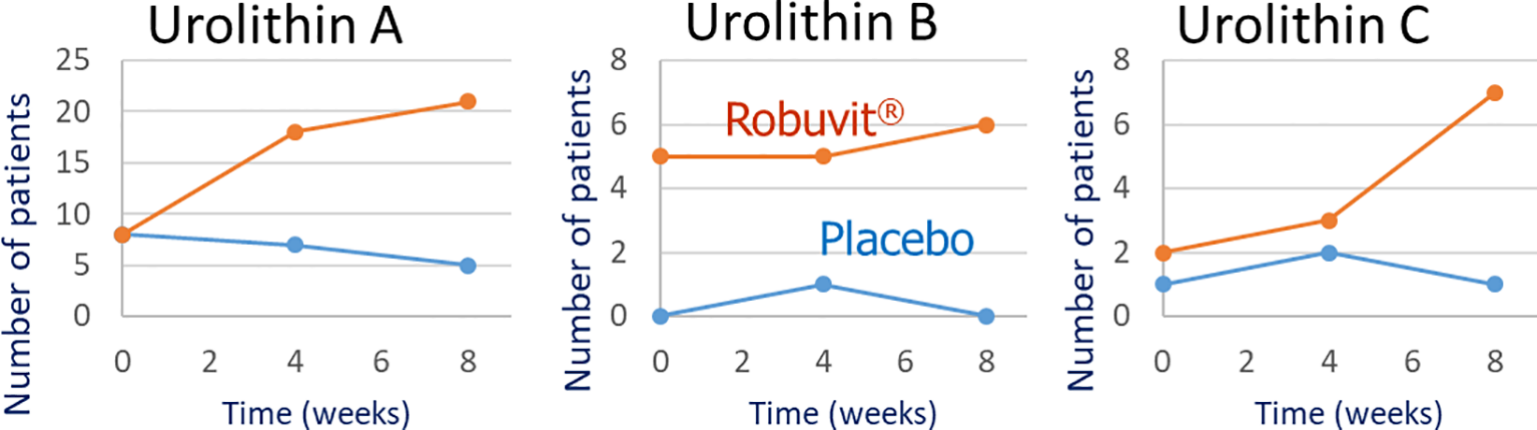
Number of patients in the Robuvit^®^ and placebo group with detectable urolithin (A–C) concentrations in serum samples from baseline (t= 0) until the end of the study (t= 8).

When the number of patients with detectable concentrations of urolithins in serum was evaluated, distinct trends were observed (**Figure 4**). Over the study period, increasingly more patients in the Robuvit^®^ group produced urolithin A (8, 18, and 21 at t = 0, t = 4, and t = 8, respectively; individual data in **Supplementary Table S10**), urolithin B (5, 5, and 6 at t = 0, t = 4, and t = 8, respectively), and urolithin C (2, 3, and 7 at t = 0, t = 4, and t = 8, respectively). In contrast, in the placebo group were fewer producers of urolithin A (8, 7, and 5 at t = 0, t = 4, and t = 8, respectively; individual data in **Supplementary Table S10**), urolithin B (0, 1, and 0 at t = 0, t = 4, and t = 8, respectively), and urolithin C (1, 2, and 1 at t = 0, t = 4, and t = 8, respectively). Analogous results were seen for urolithins in blood cells (**Supplementary Table S11**).

### Correlation of Urolithin Metabolic Phenotypes With Clinical Parameters

**Figure 5:**
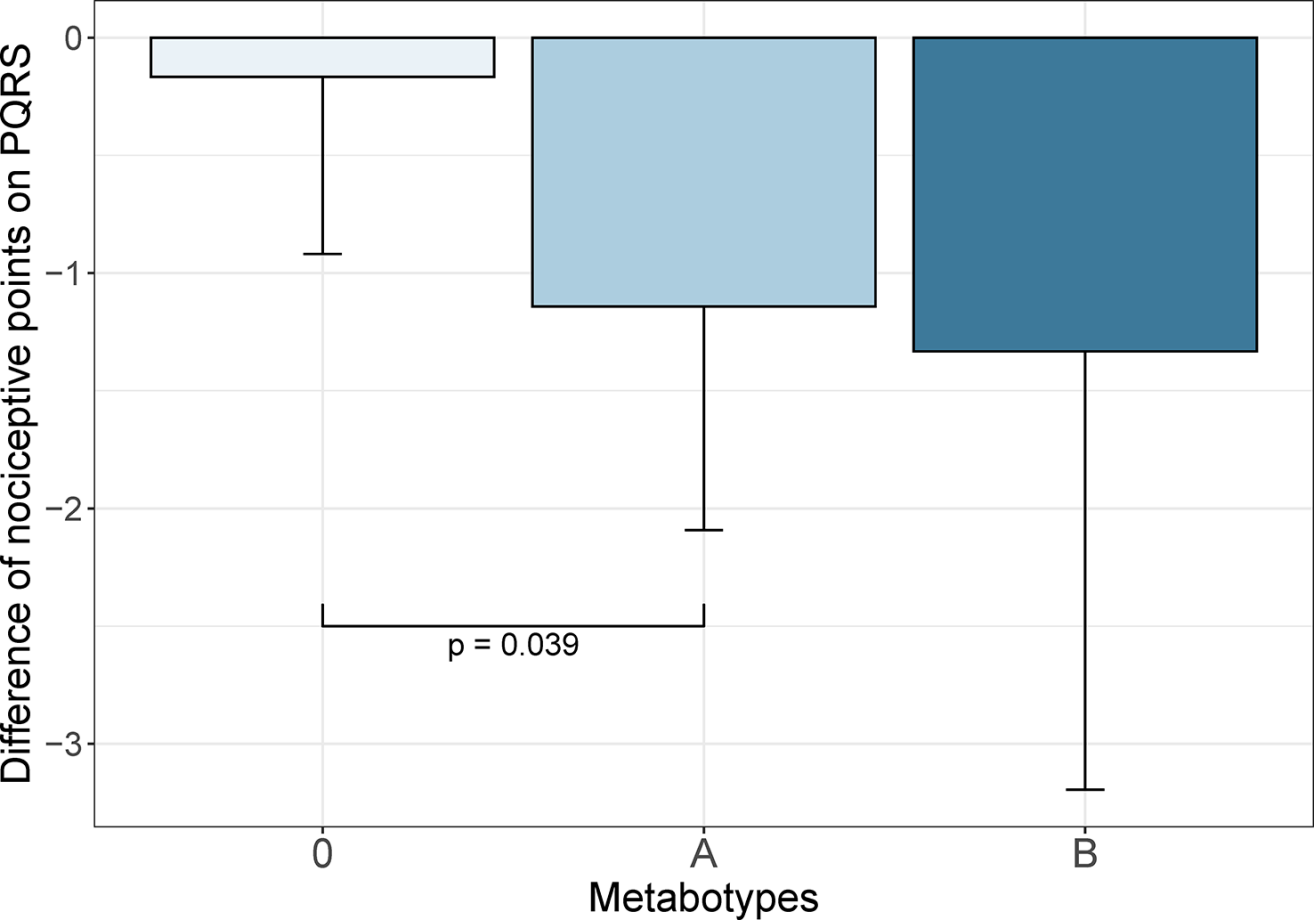
Decline of the nociceptive score points during the post-operative recovery of 26 patients who both completed the Post-operative Quality Recovery Scale (PQRS; [26]) questionnaire and blood samples were available. Based on urolithin metabolic phenotype classified in serum samples, patients with metabotype A experienced a statistically significant improvement in the pain score compared to metabotype 0 (p = 0.039). The columns represent means and standard deviation.

Both blood samples and complete Post-operative Quality Recovery Scale (PQRS) questionnaires (Royse et al., 2010) were available for 26 patients. For the Robuvit^®^ group, metabotypes were classified based on the presence of urolithins in serum. A correlation analysis between the metabolic phenotypes and changes in questionnaire items revealed a statistically significant association between the improvement of post-operative nociceptive points and the metabotype A (**Figure 5**).

The mean reduction of nociceptive points was minimal [−0.17, standard deviation (SD) 0.75] in patients with metabotype 0 (n = 6). In contrast, patients with metabotype A reported a mean decline of −1.14 (SD 0.95; n = 14) which differed by 0.98 score points (95% CI 0.05–1.90) from metabotype 0 and was statistically significantly lower (p = 0.039). The mean improvement of patients with metabotype B was even more pronounced (−1.33, SD 1.86; n = 6), but not statistically significantly different from metabotype 0 due to high interindividual data variability.

When metabotypes were classified based on the presence of urolithins in blood cells, similar results were observed. The mean reduction of nociceptive points was low (−0.30, SD 0.82) in patients with metabotype 0. Patients with metabotype A reported a mean decline of −1.40 (SD 0.84) which differed by 1.1 score points (95% CI 0.32–1.88) from metabotype 0 and was statistically significantly lower (p = 0.009). The mean improvement of patients with metabotype B was −1.33 (SD 1.86), which was not statistically significantly different from metabotype 0.

## Discussion

In the present study, oak wood constituents and metabolites were determined in serum and blood cell samples of patients before and during the recovery phase after hysterectomy. For the first time, the concentrations and distribution of urolithins between serum and blood cells was analyzed and a statistically significant association between the urolithin metabotype and post-surgical pain scores was discovered.

A sensitive analytical method allowing for the quantification of 16 different polyphenols occurring in oak wood or their metabolites was developed and validated. Thus, the determined concentrations were reliable and allowed for broad retrieval of polyphenolic compounds in patient specimen. A previously published pilot investigation with three volunteers who ingested 300 mg Robuvit^®^ focused on the detection of ellagic acid and urolithins in plasma samples. Compounds were quantified as ellagic acid equivalents due to the lack of reference standards for urolithin glucuronides (Natella et al., 2014). In the present study, analytes were quantified as after enzymatic hydrolysis of conjugates and an exploratory approach scanning for urolithin aglycones indicated a degree of conjugation close to 100%. This is consistent with previous reports (Tomas-Barberan et al., 2017).

In the patients' serum samples, 14 different polyphenols were determined. In contrast to the previous report (Natella et al., 2014), no ellagic acid was detectable above the lower limit of quantification. This was most probably due to the longer interval between the last intake of Robuvit^®^ capsules and the blood sampling in the present study (roughly 12 h) compared the earlier investigation (1 h). This suggests a rapid systemic elimination of ellagic acid, which has indeed been observed in human studies (Rios et al., 2018).

The study patients were not advised to keep a particular diet low in polyphenols. This explains why the majority of analytes were present in the serum samples of almost all participants in both study groups. In addition, many polyphenolic compounds were detected at similar concentrations before and during the intervention. Two conclusions can be derived from this fact. Firstly, a presumably mixed regular diet apparently leads to relatively high serum levels of a multitude of polyphenols such as scopoletin or vanillic acid. This calls for cautious data interpretation if nutritional studies do not include a placebo group. Secondly, the chosen study setting obviously mirrored real-life conditions with no dietary restrictions or plans to be followed. This implies both a higher complexity and validity of the acquired data and emphasizes the relevance of any differences observed between the study groups.

Despite of the long interval between the last intake of the oak wood extract and the blood sampling, the gut microbial metabolites of ellagic acid, the urolithins, were determined in serum samples. Urolithin concentrations in serum were consistently higher after intake of Robuvit^®^ capsules while this was not clearly seen in the placebo group. Due to pronounced inter-individual variability, concentrations were not statistically significantly different between the study groups. In the Robuvit^®^ group, after 8 weeks highest median concentrations of urolithin B (22.3 ng/ml) were determined, followed by urolithin A (14.0 ng/ml) and urolithin C (2.66 ng/ml). This corresponded to mean serum levels of 451 nM (urolithin A), 153 nM (urolithin B), and 26 nM (urolithin C). Compared to other ellagitannin sources such as pomegranate, strawberries, or raspberries, the urolithin concentrations observed in the present study were comparatively high in relation to the administered dose (**Table 4**), suggesting abundant formation and absorption of urolithins from oak wood extract.

**Table 4:** Comparison of mean urolithin concentrations determined in serum/plasma samples of humans after ingestion of different sources of ellagitannins.

Source	Urolithin A [nM]	Urolithin B [nM]	Urolithin C [nM]	Dose per day	Headcount	Reference
Oak wood extract	451	153	26	300 mg	n = 33	Present study
Pomegranate juice	110	50	n.d.	180 ml	n = 7	(Seeram et al., 2006 **)
Pomegranate extracts (PE-1 and PE-2)	609 132	308 10	n.d.	900 mg	n = 26	(Nunez-Sanchez et al., 2014)
Strawberries (freeze-dried)	1,964	646	n.d.	24 g	n = 19	(Sandhu et al., 2018)
Raspberries (fresh)	474	23	n.d.	300 g	n = 9	(Ludwig et al., 2015)

In serum samples, no urolithin D was detected which is consistent with observations in other studies (Tomas-Barberan et al., 2017). Only one study reported high concentrations of urolithin D in plasma (Pfundstein et al., 2014).

To the best of our knowledge, urolithin concentrations have not been quantified in blood cells before. In the present study, urolithin levels were generally lower in blood cells as compared to serum. No urolithin C was determined in any blood cell sample and urolithin B was not found in samples of the placebo group. After 8 weeks of Robuvit^®^ intake, similar median urolithin A (2.21 ng/g) and urolithin B (2.89 ng/g) levels were quantified in blood cells. These concentrations are comparable to values of 2 ng/g reported for prostate tissue samples (Tomas-Barberan et al., 2017). As explained above, in the present study urolithins were quantified after preceding enzymatic deconjugation. It can be assumed that urolithins in blood cells were mainly conjugated to glucuronic acid as reported for other specimen such as prostate tissue (Tomas-Barberan et al., 2017). This, however, raises the question how the urolithins get into blood cells or tissues. It is not clear whether conjugates of polyphenolic compounds pass cell membranes or are locally deconjugated, diffuse into the cells and undergo intracellular reconjugation (Kawai, 2018; Vinson, 2019).

Analysis of the distribution of oak wood extract constituents and metabolites between blood cells and serum revealed that urolithins were not enriched in blood cells. In contrast, ferulic acid, scopoletin, and vanillic acid preferentially distributed into blood cells. While no comparable data are available for scopoletin and vanillic acid, we previously observed a preferential distribution of ferulic acid into synovial fluid and lower concentrations in blood cells and serum (Mülek and Högger, 2015). Enrichment of compounds in specimen other than serum might contribute to a longer *in vivo* residence time and prolonged effects.

Since urolithins are products of the gut microbial metabolism of ellagitannin sources, the individual composition of the gut microbiota governs the extent of their metabolic generation. This explains the high inter-individual variability of urolithin blood concentrations observed in the present investigation. It is well known that the intestinal microbiota plays an important role in mediating physiological effects of dietary polyphenols (Kawabata et al., 2019) and that not all humans are urolithin producers (Tomas-Barberan et al., 2017). Research efforts focus on linking the composition of the gut microbiome to urolithin metabotyes as well as to cardiometabolic risk factors (Romo-Vaquero et al., 2019). In the present study population, 75% of the participants produced at least one urolithin after 8 weeks intake of Robuvit^®^. In other studies, urolithins were detected in plasma samples of 50–100% of the participants whose number ranged from 3 to 19 (Mertens-Talcott et al., 2006; Natella et al., 2014; Nunez-Sanchez et al., 2014; Ludwig et al., 2015; Sandhu et al., 2018). Interestingly, apparently the number of urolithin producers increased when higher amounts of metabolic precursors such as ellagic acid were ingested (Nunez-Sanchez et al., 2014). This supports the idea that polyphenols have prebiotic properties (Kawabata et al., 2019) and that prolonged exposure to ellagitannins or ellagic acid increases the number of urolithin producers (Tomas-Barberan et al., 2017). In the present investigation, the number of urolithin producers considerably increased over time in the Robuvit^®^, but not in the placebo group. This observation is suggestive for prebiotic properties of the oak wood extract. Additionally, it most likely mirrored the regeneration of the intestinal microbiota after surgery and the concomitant antibiotic treatment.

Based on the ability of urolithin production, three metabotypes have been proposed with the metabotype A being the most prevalent in healthy individuals (Tomas-Barberan et al., 2017). Urolithin metabotypes have been previously correlated with cardiometabolic risk biomarkers (Selma et al., 2018). In the present study, we analyzed the relation of the patients' metabotypes with clinical parameters and detected a statistically significant association of lower pain scores in patients with metabotype A. This observation was valid when assigning metabotypes based on urolithin presence both in serum (p = 0.039) and blood cells (p = 0.009). Nociceptive scoring was also lower in patients with metabotype B compared to metabotype 0, but the high data variability did not allow for a statistical confirmation. It is possible that patients producing urolithins experienced less post-operative pain and thus faster recovery due to the anti-inflammatory effects of urolithins (Tomas-Barberan et al., 2017). In cell culture models, urolithin A attenuated for example prostaglandin E_2_ production, inhibited NF-κB activity and downregulated cyclo-oxygenase-2 (COX-2) expression (Gonzalez-Sarrias et al., 2010). Anti-inflammatory effects of urolithins are consistent with the report that 50% of the patients in the placebo group as compared to 29% of the patients in the Robuvit^®^ group needed analgesics during the recovery period (Ferianec et al., 2019).

The present study has some limitations. Drop-out rates were rather high (48% in the placebo group and 27% in the Robuvit^®^ group from randomization to week 8) so that the sample number available for analysis was too low and data variability too high to confirm statistically significant differences in urolithin concentrations between the study groups. Isourolithin A was not quantified in this study. The characterisation of the study participants' metabotypes could have been refined with this additional information, since isourolithin A is another biomarker of metabotype B (Tomas-Barberan et al., 2014). Although a statistically significant correlation between the urolithin metabotype and post-operative recovery scores were observed, this has to be interpreted with caution. As a matter of course, correlations do not imply causality. It cannot be excluded that urolithin production was solely an indication for intestinal microbiota recovering from antibiotics-induced dysbiosis and that other microbiota-derived compounds exerted anti-inflammatory effects that resulted in lower pain scores. Yet, these positive results should prime further investigations and might ignite new directions of future research.

## Conclusions

While many participating patients had relatively high basal polyphenol blood concentrations, probably originating from various dietary sources, the number of urolithin producers as well as the urolithin levels consistently increased only after intake of the standardized oak wood extract Robuvit^®^. In contrast to other polyphenolic compounds, urolithins were not enriched in blood cells. The statistical significant association of the urolithin metabotype and a post-operative recovery score suggested health advantages for urolithin-producers. Further investigations have to confirm these observations and to clarify remaining open questions.

## Data Availability Statement

All datasets generated for this study are included in the article/**Supplementary Material**.

## Ethics Statement

The studies involving human participants were reviewed and approved by the Ethics Committee of the University Hospital and the Faculty of Medicine, Comenius University Bratislava (10.01.2011). The patients/participants provided their written informed consent to participate in this study.

## Author Contributions

ZD and PH contributed conception and design of the study. LV and OS-C developed the analytical method, performed the blood sample and data analysis. VF, MJ, and ZD handled the patients, collected and analyzed clinical patient data. PH wrote the first draft of the manuscript. LV, ZD, and OS-C wrote sections of the manuscript. All authors contributed to manuscript revision, read and approved the submitted version.

## Funding

This research project was funded by an educational grant of Horphag Research, Geneva, Switzerland. This publication was funded by the German Research Foundation (DFG) and the University of Würzburg in the funding program Open Access Publishing.

## Conflict of Interest

ZD and PH received an educational grant of Horphag Research, Geneva, Switzerland. The funders had no role in the design of the study; in the collection, analyses, or interpretation of data; in the writing of the manuscript, or in the decision to publish the results. 

The remaining authors declare that the research was conducted in the absence of any commercial or financial relationships that could be construed as a potential conflict of interest.
