# Supplementary material for: Constituents and Metabolites of a French Oak Wood Extract (Robuvit®) in Serum and Blood Cell Samples of Women Undergoing Hysterectomy
Source: Front Pharmacol. 2020 Feb 26;11:74. doi: 10.3389/fphar.2020.00074 (PMC7054277; doi:10.3389/fphar.2020.00074)
Supplement: Supplementary file 1 [file Table_1.docx]

Supplementary Materials

**Table S1**. LC-ESI-MS/MS settings

| Compound | Precursor ion [m/z] | Product ion [m/z] | FV [V] | CE [V] | CAV [V] | Polarity |
| --- | --- | --- | --- | --- | --- | --- |
| 1,2,3‑13C3‑ferulic acid (IS) | 196 | 136 | 100 | 13 | 5 | negative |
| (+)-Catechin | 289 | 245 | 140 | 12 | 6 | negative |
| Coniferyl aldehyde | 179 | 91 | 100 | 28 | 0 | positive |
| Ellagic acid | 301 | 145 | 140 | 42 | 0 | negative |
| Ferulic acid | 193 | 178 | 90 | 10 | 2 | negative |
| Gallic acid | 169 | 125 | 110 | 10 | 4 | negative |
| Caffeic acid | 179 | 134 | 100 | 28 | 8 | negative |
| para-Coumaric acid | 163 | 119 | 90 | 10 | 4 | negative |
| Protocatechuic acid | 153 | 108 | 100 | 24 | 4 | negative |
| Scopoletin | 193 | 133 | 130 | 20 | 5 | positive |
| Sinapic aldehyde | 209 | 55 | 110 | 12 | 5 | positive |
| Syringic aldehyde | 183 | 95 | 90 | 16 | 1 | positive |
| Syringic acid | 197 | 182 | 100 | 8 | 4 | negative |
| Urolithin A | 227 | 198 | 150 | 34 | 2 | negative |
| Urolithin B | 211 | 167 | 160 | 22 | 0 | negative |
| Urolithin C | 243 | 187 | 180 | 23 | 4 | negative |
| Urolithin D | 259 | 213 | 160 | 30 | 4 | negative |
| Vanillin | 153 | 65 | 90 | 25 | 1 | positive |
| Vanillic acid | 167 | 152 | 80 | 9 | 2 | negative |

Multiple reaction monitoring transitions selected for quantification and their respective optimized fragmentor voltages (FV), collision energies (CE), cell acceleration voltages (CAV) and polarities.

**Table S2 a.** Validation results for interday precision and accuracy in human pooled serum (n=15).

| **Analyte** | **Spiked concentration [ng/mL]** | **Measured concentration** | | |
| --- | --- | --- | --- | --- |
|  |  | Mean concentration ± SD [ng/mL] | Accuracy [%] | Precision [%] |
| Urolithin A | 0.94 | 0.98 ± 0.11 | 104.17 | 10.76 |
|  | 1.89 | 2.02 ± 0.12 | 106.98 | 5.84 |
|  | 9.43 | 9.79 ± 0.72 | 103.83 | 7.41 |
|  | 23.57 | 24.60 ± 1.49 | 104.40 | 6.07 |
|  | 47.13 | 49.17 ± 1.54 | 104.33 | 3.14 |
| Urolithin B | 0.45 | 0.47 ± 0.06 | 102.62 | 12.47 |
|  | 0.91 | 0.93 ± 0.06 | 102.47 | 6.16 |
|  | 4.55 | 4.31 ± 0.49 | 94.71 | 11.30 |
|  | 11.36 | 11.39 ± 0.66 | 100.62 | 5.43 |
|  | 22.73 | 22.37 ± 1.43 | 98.40 | 6.38 |
| Urolithin C | 0.73 | 0.76 ± 0.08 | 104.94 | 10.76 |
|  | 1.45 | 1.45 ± 0.15 | 100.12 | 10.61 |
|  | 7.25 | 6.50 ± 0.39 | 89.66 | 5.93 |
|  | 18.13 | 17.73 ± 0.96 | 97.80 | 5.41 |
|  | 36.26 | 37.90 ± 1.20 | 104.53 | 3.16 |
| Urolithin D | 0.95 | 1.08 ± 0.13 | 113.38 | 12.45 |
|  | 1.90 | 2.01 ± 0.22 | 106.03 | 10.69 |
|  | 9.50 | 8.66 ± 0.74 | 91.13 | 3.00 |
|  | 23.75 | 24.10 ± 1.34 | 101.47 | 5.55 |
|  | 47.50 | 48.94 ± 1.86 | 103.04 | 3.80 |
| Syringic acid | 4.71 | 4.72 ± 0.91 | 100.07 | 19.31 |
|  | 9.43 | 9.47 ± 1.39 | 100.45 | 14.70 |
|  | 47.13 | 44.87 ± 4.49 | 95.19 | 10.01 |
|  | 117.83 | 115 ± 3.73 | 97.60 | 98.63 |
|  | 235.66 | 232.43 ± 10.71 | 98.63 | 4.61 |
| Ferulic acid | 1.91 | 1.98 ± 0.22 | 103.53 | 11.37 |
|  | 3.82 | 3.79 ± 0.35 | 99.10 | 9.23 |
|  | 19.10 | 19.20 ± 1.31 | 100.51 | 6.81 |
|  | 47.75 | 47.23 ± 2.54 | 98.90 | 5.37 |
|  | 95.51 | 90.70 ± 3.53 | 94.97 | 3.89 |

**Table S2a** *(continued).*

| **Analyte** | **Spiked concentration [ng/mL]** | **Measured concentration** | | |
| --- | --- | --- | --- | --- |
|  |  | Mean concentration ± SD [ng/mL] | Accuracy [%] | Precision [%] |
| Caffeic acid | 0.96 | 0.97 ± 0.17 | 101.75 | 17.74 |
|  | 1.91 | 1.92 ± 0.17 | 100.30 | 8.76 |
|  | 9.56 | 9.70 ± 0.98 | 101.49 | 10.07 |
|  | 23.90 | 24.16 ± 1.41 | 101.10 | 5.83 |
|  | 47.80 | 50.03 ± 1.92 | 104.66 | 3.83 |
| Gallic acid | 4.37 | 4.44 ± 0.51 | 101.52 | 11.43 |
|  | 8.54 | 8.33 ± 0.51 | 97.61 | 6.14 |
|  | 43.72 | 41.10 ± 2.41 | 93.99 | 5.85 |
|  | 109.31 | 105.31 ± 4.76 | 96.34 | 4.52 |
|  | 213.42 | 213.34 ± 4.85 | 99.96 | 2.27 |
| Vanillic acid | 17.21 | 15.98 ± 2.72 | 92.86 | 16.99 |
|  | 34.42 | 33.15 ± 2.59 | 96.30 | 7.80 |
|  | 172.12 | 164.32 ± 13.88 | 95.47 | 8.45 |
|  | 430.29 | 433.14 ± 15.96 | 100.66 | 3.68 |
|  | 860.58 | 931.63 ± 31.37 | 108.26 | 3.37 |
| Para-Coumaric acid | 0.96 | 0.93 ± 0.16 | 97.56 | 17.01 |
|  | 1.91 | 1.93 ± 0.15 | 101.22 | 7.85 |
|  | 9.55 | 9.49 ± 0.49 | 99.35 | 5.19 |
|  | 23.88 | 23.87 ± 0.94 | 99.98 | 3.93 |
|  | 47.75 | 49.10 ± 1.55 | 102.83 | 3.02 |
| Protocatechuic acid | 12.58 | 11.67 ± 1.10 | 92.74 | 9.45 |
|  | 25.16 | 27.17 ± 2.84 | 107.99 | 10.45 |
|  | 125.81 | 125.64 ± 8.69 | 99.87 | 6.92 |
|  | 314.53 | 310.69 ± 17.63 | 98.78 | 5.67 |
|  | 629.07 | 622.93 ± 32.57 | 99.02 | 5.23 |
| Sinapinaldehyde | 0.94 | 0.85 ± 0.11 | 90.59 | 12.90 |
|  | 1.88 | 1.85 ± 0.16 | 98.44 | 8.72 |
|  | 9.38 | 8.96 ± 0.34 | 95.53 | 3.75 |
|  | 23.45 | 23.24 ± 1.30 | 99.11 | 5.61 |
|  | 46.89 | 46.52 ± 1.22 | 99.20 | 2.62 |

**Table S2a.** *(continued)*

| **Analyte** | **Spiked concentration [ng/mL]** | **Measured concentration** | | |
| --- | --- | --- | --- | --- |
|  |  | Mean concentration ± SD [ng/mL] | Accuracy [%] | Precision [%] |
| Scopoletin | 6.32 | 5.51 ± 0.74 | 87.19 | 17.89 |
|  | 12.63 | 12.79 ± 1.47 | 101.25 | 11.47 |
|  | 63.16 | 63.85 ± 3.05 | 101.08 | 5.33 |
|  | 157.91 | 160.06 ± 6.40 | 101.37 | 4.00 |
|  | 315.81 | 296.01 ± 8.26 | 93.73 | 2.79 |
| Syringaldehyde | 0.32 | 0.32 ± 0.06 | 100.36 | 17.50 |
|  | 0.63 | 0.65 ± 0.07 | 103.17 | 10.61 |
|  | 3.17 | 3.08 ± 0.22 | 97.08 | 7.12 |
|  | 7.92 | 8.27 ± 0.32 | 104.38 | 3.81 |
|  | 15.84 | 16.68 ± 0.41 | 105.31 | 2.47 |
| Coniferaldehyde | 0.62 | 0.64 ± 0.09 | 102.13 | 14.37 |
|  | 1.25 | 1.33 ± 0.12 | 106.59 | 8.68 |
|  | 6.25 | 6.03 ± 0.40 | 96.47 | 6.67 |
|  | 15.62 | 16.62 ± 1.17 | 106.44 | 7.04 |
|  | 31.23 | 33.53 ± 1.29 | 107.37 | 3.85 |
| Vanillin | 1.91 | 2.07 ± 0.34 | 108.10 | 16.37 |
|  | 3.83 | 3.90 ± 0.46 | 101.86 | 11.38 |
|  | 19.13 | 17.82 ± 1.44 | 93.12 | 8.08 |
|  | 47.84 | 47.08 ± 3.80 | 98.41 | 8.06 |
|  | 95.67 | 93.35 ± 5.38 | 97.57 | 5.76 |

**Table S2 b.** Validation results for interday precision and accuracy in human pooled blood cells (n=15).

| **Analyte** | **Spiked concentration [ng/g]** | **Measured concentration** | | |
| --- | --- | --- | --- | --- |
|  |  | Mean concentration ± SD [ng/g] | Accuracy [%] | Precision [%] |
| Urolithin A | 0.97 | 1.00 ± 0.06 | 102.39 | 6.34 |
|  | 1.95 | 1.91 ± 0.14 | 98.00 | 7.07 |
|  | 4.88 | 4.82 ± 0.23 | 98.68 | 4.73 |
|  | 6.51 | 6.37 ± 0.47 | 97.72 | 7.40 |
|  | 10.88 | 10.91 ± 1.04 | 100.23 | 9.53 |
| Urolithin B | 0.63 | 0.63 ± 0.03 | 100.76 | 4.15 |
|  | 1.25 | 1.22 ± 0.07 | 97.54 | 5.43 |
|  | 3.14 | 3.12 ± 0.08 | 99.29 | 2.53 |
|  | 4.19 | 4.18 ± 0.23 | 99.77 | 5.44 |
|  | 7.00 | 6.85 ± 0.41 | 97.89 | 5.95 |
| Urolithin C | 3.23 | 3.11 ± 0.12 | 96.36 | 3.98 |
|  | 6.47 | 6.10 ± 0.32 | 94.32 | 5.17 |
|  | 16.19 | 14.12 ± 0.47 | 87.20 | 5.68 |
|  | 21.60 | 20.39 ± 1.19 | 94.41 | 5.84 |
|  | 36.08 | 33.01 ± 3.24 | 91.50 | 9.80 |
| Urolithin D | 4.70 | 4.06 ± 0.62 | 86.31 | 15.29 |
|  | 9.40 | 9.34 ± 1.31 | 99.35 | 14.03 |
|  | 23.54 | 21.47 ± 3.00 | 91.20 | 13.98 |
|  | 31.41 | 30.60 ± 2.65 | 97.43 | 8.67 |
|  | 52.47 | 50.79 ± 6.60 | 96.81 | 13.00 |
| Syringic acid | 14.61 | 14.40 ± 1.34 | 98.52 | 9.79 |
|  | 29.25 | 28.21 ± 2.58 | 96.42 | 9.13 |
|  | 73.23 | 72.96 ± 2.51 | 99.63 | 3.44 |
|  | 97.71 | 97.88 ± 3.68 | 100.18 | 3.76 |
|  | 163.20 | 163.85 ± 6.33 | 100.40 | 3.86 |
| Ferulic acid | 4.94 | 4.81 ± 0.32 | 97.48 | 6.83 |
|  | 9.88 | 10.05 ± 0.59 | 101.70 | 5.88 |
|  | 24.73 | 24.66 ± 0.47 | 99.72 | 1.92 |
|  | 33.00 | 32.91 ± 1.19 | 99.74 | 3.61 |
|  | 55.12 | 54.91 ± 3.01 | 99.63 | 5.49 |

**Table S2b** *(continued).*

| **Analyte** | **Spiked concentration [ng/g]** | **Measured concentration** | | |
| --- | --- | --- | --- | --- |
|  |  | Mean concentration ± SD [ng/g] | Accuracy [%] | Precision [%] |
| Caffeic acid | 16.47 | 16.42 ± 1.02 | 99.71 | 6.20 |
|  | 32.97 | 33.10 ± 1.23 | 100.41 | 3.73 |
|  | 82.52 | 84.21 ± 8.76 | 102.04 | 10.40 |
|  | 110.11 | 108.55 ± 4.65 | 98.58 | 4.28 |
|  | 183.91 | 185.89 ± 11.80 | 101.08 | 6.35 |
| Gallic acid | 9.68 | 9.27 ± 0.75 | 95.73 | 8.07 |
|  | 19.38 | 19.99 ± 1.13 | 103.13 | 6.57 |
|  | 48.52 | 47.71 ± 5.21 | 98.33 | 10.92 |
|  | 64.74 | 64.91 ± 3.67 | 100.27 | 5.66 |
|  | 108.14 | 105.84 ± 8.69 | 97.88 | 8.21 |
| Vanillic acid | 24.26 | 25.45 ± 4.91 | 104.89 | 19.30 |
|  | 48.56 | 48.44 ± 2.94 | 99.76 | 6.06 |
|  | 121.55 | 120.65 ± 7.32 | 99.25 | 6.07 |
|  | 162.18 | 161.39 ± 4.76 | 99.51 | 2.95 |
|  | 270.89 | 274.56 ± 5.67 | 101.35 | 2.07 |
| Para-Coumaric acid | 2.63 | 2.77 ± 0.12 | 105.31 | 4.29 |
|  | 5.27 | 5.29 ± 0.15 | 100.42 | 2.86 |
|  | 13.19 | 13.01 ± 0.34 | 98.63 | 2.61 |
|  | 17.60 | 17.61 ± 0.27 | 100.05 | 1.56 |
|  | 29.40 | 30.57 ± 1.15 | 104.00 | 3.75 |
| Scopoletin | 8.16 | 8.54 ± 0.96 | 104.62 | 11.23 |
|  | 16.33 | 15.52 ± 1.34 | 94.99 | 8.66 |
|  | 40.89 | 40.54 ± 2.26 | 99.14 | 5.57 |
|  | 54.56 | 56.19 ± 4.34 | 103.00 | 7.72 |
|  | 91.13 | 90.86 ± 7.39 | 99.71 | 8.14 |

**Table S3 a.** Validation results for recovery in human pooled serum (n=5).

| **Analyte** | **Spiked concentration [ng/mL]** | **Recovery** | **Process efficiency** |
| --- | --- | --- | --- |
|  |  | Mean ± SD [%] | Mean ± SD [%] |
| Urolithin A | 1.89 | 92.5 ± 1.62 | 88.4 ± 5.11 |
|  | 47.13 | 93.7 ± 1.75 | 86.1 ± 4.17 |
| Urolithin B | 0.91 | 90.0 ± 1.58 | 92.6 ± 13.18 |
|  | 22.73 | 89.4 ± 1.19 | 103.3 ± 4.09 |
| Urolithin C | 1.45 | 88.1 ± 3.46 | 111.3 ± 2.26 |
|  | 36.26 | 86.3 ± 1.98 | 125.3 ± 1.89 |
| Urolithin D | 1.90 | 93.3 ± 1.71 | 103.0 ± 1.48 |
|  | 47.50 | 93.2 ± 1.91 | 100.6 ± 0.93 |
| Syringic acid | 9.43 | 91.9 ± 4.16 | 136.5 ± 18.27 |
|  | 235.66 | 90.6 ± 0.62 | 124.6 ± 8.58 |
| Ferulic acid | 3.82 | 94.4 ± 2.63 | 85.2 ± 2.63 |
|  | 95.51 | 93.1 ± 1.10 | 96.2 ± 1.35 |
| Caffeic acid | 1.91 | 66.1 ± 4.02 | 108.9 ± 6.13 |
|  | 47.80 | 64.8 ± 1.14 | 112.6 ± 1.52 |
| Gallic acid | 8.54 | 83.9 ± 16.73 | 85.8 ± 16.73 |
|  | 213.42 | 81.7 ± 6.48 | 93.9 ± 8.82 |
| Vanillic acid | 34.42 | 87.7 ± 6.11 | 153.1 ± 3.86 |
|  | 860.58 | 88.3 ± 0.68 | 149.0 ± 13.24 |
| Para-coumaric acid | 1.91 | 96.3 ± 3.58 | 93.4 ± 5.15 |
|  | 47.75 | 94.4 ± 1.50 | 101.4 ± 1.26 |
| Protocatechuic acid | 25.16 | 82.9 ± 0.59 | 102.7 ± 14.44 |
|  | 629.07 | 84.3 ± 1.19 | 102.2 ± 8.93 |
| Sinapinaldehyde | 1.88 | 55.7 ± 0.60 | 53.4 ± 2.30 |
|  | 46.89 | 57.0 ± 1.49 | 52.5 ± 2.47 |
| Scopoletin | 12.63 | 92.9 ± 1.91 | 86.1 ± 2.02 |
|  | 315.81 | 92.6 ± 0.51 | 85.2 ± 4.26 |
| Syringaldehyde | 0.63 | 72.9 ± 9.55 | 70.4 ± 4.30 |
|  | 15.84 | 72.3 ± 2.03 | 71.1 ± 1.46 |

**Table S3a** *(continued).*

| **Analyte** | **Spiked concentration [ng/mL]** | **Recovery** | **Process efficiency** |
| --- | --- | --- | --- |
|  |  | Mean ± SD [%] | Mean ± SD [%] |
| Coniferaldehyde | 1.25 | 60.6 ± 2.57 | 56.7 ± 2.57 |
|  | 31.23 | 58.8 ± 0.40 | 53.6 ± 2.36 |
| Vanillin | 3.83 | 45.6 ± 2.19 | 40.7 ± 2.19 |
|  | 95.67 | 45.1 ± 1.83 | 41.1 ± 1.21 |

**Table S3 b.** Validation results for recovery in human pooled blood cells (n=5).

| **Analyte** | **Spiked concentration [ng/g]** | **Recovery** | **Process efficiency** |
| --- | --- | --- | --- |
|  |  | Mean ± SD [%] | Mean ± SD [%] |
| Urolithin A | 1.95 | 69.4 ± 1.43 | 69.7 ± 2.06 |
|  | 10.88 | 72.1 ± 0.70 | 65.3 ± 2.44 |
| Urolithin B | 1.25 | 78.6 ± 0.85 | 99.4 ± 3.54 |
|  | 7.00 | 79.2 ± 2.04 | 84.0 ± 10.64 |
| Urolithin C | 6.47 | 39.3 ± 2.78 | 37.7 ± 9.70 |
|  | 36.08 | 37.2 ± 1.33 | 33.6 ± 4.75 |
| Urolithin D | 9.40 | 3.9 ± 1.38 | 4.1 ± 1.50 |
|  | 52.47 | 4.0 ± 0.95 | 4.0 ± 1.31 |
| Syringic acid | 29.25 | 47.1 ± 4.42 | 74.9 ± 9.91 |
|  | 163.20 | 45.3 ± 1.14 | 80.2 ± 1.14 |
| Ferulic acid | 9.88 | 81.6 ± 3.74 | 75.3 ± 5.90 |
|  | 55.12 | 81.1 ± 1.72 | 81.8 ± 4.81 |
| Caffeic acid | 32.97 | 14.3 ± 1.93 | 17.2 ± 2.39 |
|  | 183.91 | 14.2 ± 0.67 | 16.5 ± 1.59 |
| Gallic acid | 19.38 | 3.9 ± 0.22 | 4.9 ± 0.30 |
|  | 108.14 | 2.8 ± 0.26 | 3.1 ± 0.23 |
| Vanillic acid | 48.56 | 47.4 ± 7.59 | 134.1 ± 16.92 |
|  | 270.89 | 47.4 ± 5.30 | 110.4 ± 12.64 |
| Para-coumaric acid | 5.27 | 47.7 ± 2.46 | 66.6 ± 5.73 |
|  | 29.40 | 49.6 ± 1.24 | 59.9 ± 2.81 |
| Scopoletin | 16.33 | 86.3 ± 1.87 | 72.1 ± 2.20 |
|  | 91.13 | 88.0 ± 1.22 | 74.5 ± 2.31 |

**Table S4 a.** Validation results for dilution integrity in human pooled serum (n=6).

| **Analyte** | **Spiked concen-tration above ULOQ [ng/mL]** | **Dilution factor** | **Measured and backcalculated concentration** | | | |
| --- | --- | --- | --- | --- | --- | --- |
|  |  |  | Concen-tration [ng/mL] | Mean [ng/mL] | Accuracy [%] ± SD | Precision [%] |
| Urolithin A | 147.75 | 1:10 | 161.70 | 160.34 | 108.5 ± 3.5 | 3.2 |
|  |  |  | 159.00 |  |  |  |
|  |  |  | 160.64 |  |  |  |
|  |  |  | 151.82 |  |  |  |
|  |  |  | 167.77 |  |  |  |
|  |  |  | 161.12 |  |  |  |
| Urolithin B | 71.25 | 1:10 | 65.53 | 66.56 | 93.4 ± 5.8 | 6.2 |
|  |  |  | 62.19 |  |  |  |
|  |  |  | 68.33 |  |  |  |
|  |  |  | 62.40 |  |  |  |
|  |  |  | 73.19 |  |  |  |
|  |  |  | 67.70 |  |  |  |

**Table S4 b.** Validation results for dilution integrity in human pooled blood cells (n=6).

| **Analyte** | **Spiked concen-tration above ULOQ [ng/mL]** | **Dilution factor** | **Measured and backcalculated concentration** | | | |
| --- | --- | --- | --- | --- | --- | --- |
|  |  |  | Concen-tration [ng/mL] | Mean [ng/mL] | Accuracy [%] ± SD | Precision [%] |
| Urolithin A | 44.30 | 1:10 | 49.88 | 47.90 | 108.11 ± 6.58 | 6.08 |
|  |  |  | 48.75 |  |  |  |
|  |  |  | 49.98 |  |  |  |
|  |  |  | 45.29 |  |  |  |
|  |  |  | 43.27 |  |  |  |
|  |  |  | 50.20 |  |  |  |
| Urolithin B | 42.73 | 1:10 | 49.43 | 47.47 | 111.10 ± 4.96 | 4.47 |
|  |  |  | 48.09 |  |  |  |
|  |  |  | 49.89 |  |  |  |
|  |  |  | 46.13 |  |  |  |
|  |  |  | 44.23 |  |  |  |
|  |  |  | 47.06 |  |  |  |

**Table S5 a.** Validation results for matrix effects in six different lots of human serum (n=3).

| **Analyte** | **Spiked concentration [ng/mL]** | **IS normalized Matrix factor of six different lots of human serum** | |
| --- | --- | --- | --- |
|  |  | Mean MF [%] | RSD [%] |
| Urolithin A | 1.89 | 107.54 | 13.74 |
|  | 47.13 | 106.47 | 8.67 |
| Urolithin B | 0.91 | 88.02 | 6.89 |
|  | 22.73 | 88.24 | 5.81 |
| Urolithin C | 1.45 | 111.73 | 7.60 |
|  | 36.26 | 134.69 | 3.36 |
| Urolithin D | 1.90 | 91.80 | 9.92 |
|  | 47.50 | 102.61 | 3.30 |
| Syringic acid | 9.43 | 121.30 | 12.07 |
|  | 235.66 | 120.62 | 4.90 |
| Ferulic acid | 3.82 | 87.18 | 7.38 |
|  | 95.51 | 96.60 | 4.30 |
| Caffeic acid | 1.91 | 115.83 | 9.58 |
|  | 47.80 | 111.19 | 5.15 |
| Gallic acid | 8.54 | 93.97 | 8.91 |
|  | 213.42 | 122.41 | 6.02 |
| Vanillic acid | 34.42 | 180.28 | 12.93 |
|  | 860.58 | 170.56 | 8.68 |
| Para-coumaric acid | 1.91 | 102.90 | 7.77 |
|  | 47.75 | 109.07 | 4.05 |
| Protocatechuic acid | 25.16 | 120.04 | 8.55 |
|  | 629.07 | 116.73 | 4.25 |
| Sinapinaldehyde | 1.88 | 92.96 | 5.92 |
|  | 46.89 | 93.10 | 4.46 |
| Scopoletin | 12.63 | 90.30 | 6.12 |
|  | 315.81 | 89.98 | 4.17 |
| Syringaldehyde | 0.63 | 101.85 | 12.82 |
|  | 15.84 | 96.97 | 6.20 |

**Table S5a.** *(continued).*

| **Analyte** | **Spiked concentration [ng/mL]** | **IS normalized Matrix factor of six different lots of human serum** | |
| --- | --- | --- | --- |
|  |  | Mean MF [%] | RSD [%] |
| Coniferaldehyde | 1.25 | 84.41 | 14.36 |
|  | 31.23 | 92.64 | 3.57 |
| Vanillin | 3.83 | 85.69 | 10.13 |
|  | 95.67 | 88.44 | 4.15 |

**Table S5 b.** Validation results for matrix effects in four different lots of human blood cells (n=3).

| **Analyte** | **Spiked concentration [ng/g]** | **IS normalized Matrix factor of four different lots of human blood cells** | |
| --- | --- | --- | --- |
|  |  | Mean MF [%] | RSD [%] |
| Urolithin A | 1.95 | 84.89 | 5.82 |
|  | 10.88 | 77.92 | 5.03 |
| Urolithin B | 1.25 | 107.19 | 4.44 |
|  | 7.00 | 87.53 | 11.19 |
| Urolithin C | 6.47 | 84.41 | 4.47 |
|  | 36.08 | 86.93 | 4.50 |
| Urolithin D | 9.40 | 96.16 | 11.35 |
|  | 52.47 | 104.78 | 9.35 |
| Syringic acid | 29.25 | 133.97 | 2.67 |
|  | 163.20 | 137.32 | 2.21 |
| Ferulic acid | 9.88 | 78.17 | 5.88 |
|  | 55.12 | 86.03 | 3.36 |
| Caffeic acid | 32.97 | 103.09 | 8.81 |
|  | 183.91 | 97.50 | 6.41 |
| Gallic acid | 19.38 | 106.37 | 10.29 |
|  | 108.14 | 102.05 | 13.12 |
| Vanillic acid | 48.56 | 238.02 | 7.22 |
|  | 270.89 | 197.25 | 6.87 |
| Para-coumaric acid | 5.27 | 116.56 | 2.38 |
|  | 29.40 | 103.74 | 3.28 |
| Scopoletin | 16.33 | 69.43 | 4.36 |
|  | 91.13 | 70.97 | 4.67 |

**Table S6 a.** Validation results for short-term stability in human pooled serum after 4 h at room temperature
(n = 5)

| **Analyte** | **Spiked concentration [ng/mL]** | **Measured concentration** | | |
| --- | --- | --- | --- | --- |
|  |  | Mean ± SD [ng/mL] | RSD [%] | Δ [%] |
| Urolithin A | 1.89 | 1.97 ± 0.11 | 5.57 | 4.58 |
|  | 47.13 | 48.67 ± 0.92 | 1.88 | 3.26 |
| Urolithin B | 0.91 | 0.92 ± 0.07 | 7.80 | 1.27 |
|  | 22.73 | 21.62 ± 1.05 | 4.95 | -4.86 |
| Urolithin C | 1.45 | 1.30 ± 0.09 | 7.11 | -10.37 |
|  | 36.26 | 41.19 ± 1.79 | 4.35 | 13.59 |
| Urolithin D | 1.90 | 2.14 ± 0.08 | 8.92 | 12.45 |
|  | 47.50 | 52.00 ± 3.02 | 5.03 | 9.48 |
| Syringic acid | 9.43 | 9.51 ± 0.72 | 7.54 | 0.91 |
|  | 235.66 | 226.70 ± 16.16 | 7.13 | -3.80 |
| Ferulic acid | 3.82 | 4.31 ± 0.71 | 16.41 | 12.86 |
|  | 95.51 | 97.86 ± 4.83 | 4.94 | 2.47 |
| Caffeic acid | 1.91 | 2.20 ± 0.33 | 15.00 | 15.30 |
|  | 47.80 | 46.12 ± 0.58 | 1.26 | -3.51 |
| Gallic acid | 8.54 | 8.40 ± 0.78 | 9.30 | -1.60 |
|  | 213.42 | 214.01 ± 8.15 | 3.81 | 0.28 |
| Vanillic acid | 34.42 | 36.49 ± 5.53 | 15.14 | 6.01 |
|  | 860.58 | 952.21 ± 20.89 | 2.19 | 10.65 |
| Para-coumaric acid | 1.91 | 2.18 ± 0.17 | 7.75 | 14.23 |
|  | 47.75 | 52.62 ± 2.57 | 4.89 | 10.18 |
| Protocatechuic acid | 25.16 | 24.81 ± 2.38 | 9.58 | -1.40 |
|  | 629.07 | 596.96 ± 71.07 | 11.91 | -5.10 |
| Sinapinaldehyde | 1.88 | 1.84 ± 0.11 | 6.04 | -1.85 |
|  | 46.89 | 47.87 ± 2.40 | 5.01 | 2.09 |
| Scopoletin | 12.63 | 13.26 ± 0.49 | 3.72 | 4.94 |
|  | 315.81 | 288.78 ± 10.99 | 3.81 | -8.56 |
| Syringaldehyde | 0.63 | 0.65 ± 0.06 | 9.78 | 3.22 |
|  | 15.84 | 15.88 ± 0.62 | 3.92 | 0.27 |

**Table S6a.** *(continued)*

| **Analyte** | **Spiked concentration [ng/mL]** | **Measured concentration** | | |
| --- | --- | --- | --- | --- |
|  |  | Mean ± SD [ng/mL] | RSD [%] | Δ [%] |
| Coniferaldehyde | 1.25 | 1.15 ± 0.06 | 5.38 | -7.96 |
|  | 31.23 | 32.64 ± 1.21 | 3.72 | 4.51 |
| Vanillin | 3.83 | 3.70 ± 0.42 | 11.38 | -3.39 |
|  | 95.67 | 96.40 ± 6.14 | 6.37 | 0.77 |

Δ = (Mean measured concentration/spiked concentration )- 1

**Table S6 b.** Validation results for short-term stability in human pooled blood cells after 4 h at room temperature (n = 5)

| **Analyte** | **Spiked concentration [ng/g]** | **Measured concentration** | | |
| --- | --- | --- | --- | --- |
|  |  | Mean ± SD [ng/g] | RSD [%] | Δ [%] |
| Urolithin A | 1.95 | 2.06 ± 0.10 | 5.02 | 5.54 |
|  | 10.88 | 11.62 ± 0.79 | 6.82 | 6.77 |
| Urolithin B | 1.25 | 1.19 ± 0.02 | 2.04 | -5.29 |
|  | 7.00 | 7.10 ± 0.36 | 5.03 | 1.50 |
| Urolithin C | 6.47 | 5.73 ± 0.07 | 1.30 | -11.45 |
|  | 36.08 | 33.87 ± 1.10 | 3.25 | -6.12 |
| Urolithin D | 9.40 | 7.34 ± 1.01 | 13.76 | -21.95 |
|  | 52.47 | 38.73 ± 2.15 | 5.55 | -26.17 |
| Syringic acid | 29.25 | 27.67 ± 2.81 | 10.16 | -5.43 |
|  | 163.20 | 161.10 ± 9.03 | 5.61 | -1.28 |
| Ferulic acid | 9.88 | 9.08 ± 0.58 | 6.38 | -8.09 |
|  | 55.12 | 54.99 ± 3.31 | 6.02 | -0.23 |
| Caffeic acid | 32.97 | 36.51 ± 3.16 | 8.67 | 10.74 |
|  | 183.91 | 171.27 ± 9.54 | 5.57 | -6.87 |
| Gallic acid | 19.38 | 21.07 ± 1.83 | 8.70 | 8.70 |
|  | 108.14 | 95.01 ± 4.91 | 5.17 | -12.14 |
| Vanillic acid | 48.56 | 51.94 ± 5.59 | 6.31 | 6.96 |
|  | 270.89 | 241.82 ± 14.99 | 6.20 | -10.73 |
| Para-coumaric acid | 5.27 | 5.52 ± 0.10 | 1.77 | 4.70 |
|  | 29.40 | 31.77 ± 1.28 | 4.04 | 8.07 |
| Scopoletin | 16.33 | 17.33 ± 1.02 | 5.86 | 6.11 |
|  | 91.13 | 96.93 ± 4.96 | 5.12 | 6.37 |

Δ = (Mean measured concentration/spiked concentration )- 1

**Table S7 a.** Validation results for linearity in human pooled serum (n = 5)

| **Analyte** | **Calibration range [ng/mL]** | **Slope ± SD** | **Y-Intercept** | **Correlation coefficient R^2^** |
| --- | --- | --- | --- | --- |
| Urolithin A | 0.94 – 103.43 | 0.0283 ± 0.0013 | -0.0030 | 0.9996 |
| Urolithin B | 0.45 – 49.88 | 0.2332 ± 0.0079 | -0.0306 | 0.9937 |
| Urolithin C | 0.63 – 34.30 | 0.0239 ± 0.0005 | 0.0036 | 0.9963 |
| Urolithin D | 0.91 – 99.75 | 0.0220 ± 0.0005 | -0.0020 | 0.9971 |
| Syringic acid | 4.71 – 517.13 | 0.0008 ± 0.00003 | -0.0007 | 0.9935 |
| Ferulic acid | 1.91 – 209.58 | 0.0056± 0.0002 | 0.0030 | 0.9955 |
| Caffeic acid | 0.96 – 52.45 | 0.0213 ± 0.0021 | 0.0001 | 0.9863 |
| Gallic acid | 4.37 – 479.71 | 0.0086 ± 0.0003 | -0.0070 | 0.9945 |
| Vanillic acid | 17.21 – 944.21 | 0.0050 ± 0.0003 | -0.0248 | 0.9949 |
| Para-coumaric acid | 0.95 – 104.79 | 0.0335 ± 0.0012 | 0.0082 | 0.9972 |
| Protocatechuic acid | 12.58 – 1380.4 | 0.0058 ± 0.0005 | -0.0200 | 0.9988 |
| Sinapinaldehyde | 0.94 – 102.90 | 0.0592 ± 0.0046 | -0.0103 | 0.9954 |
| Scopoletin | 6.31 – 173.25 | 0.5646 ± 0.0289 | -0.3173 | 0.9991 |
| Syringaldehyde | 0.32 – 34.76 | 0.1571 ± 0.0155 | -0.0194 | 0.9960 |
| Coniferaldehyde | 0.62 – 58.63 | 0.0474 ± 0.0102 | 0.0039 | 0.9922 |
| Vanillin | 1.91 – 104.97 | 0.0482 ± 0.0294 | 0.0036 | 0.9949 |

**Table S7 b.** Validation results for linearity in human pooled blood cells (n = 5)

| **Analyte** | **Calibration range [ng/mL]** | **Slope ± SD** | **Y-Intercept** | **Correlation coefficient R^2^** |
| --- | --- | --- | --- | --- |
| Urolithin A | 0.97 – 13.79 | 0.0720 ± 0.0016 | -0.0080 | 0.9991 |
| Urolithin B | 0.63 – 8.87 | 0.3662 ± 0.0072 | -0.0529 | 0.9983 |
| Urolithin C | 3.23 – 45.73 | 0.0364 ± 0.0002 | -0.2542 | 0.9913 |
| Urolithin D | 4.70 – 66.49 | 0.0030 ± 0.0001 | -0.0006 | 0.9959 |
| Syringic acid | 14.62 – 206.83 | 0.0029 ± 0.0001 | -0.0005 | 0.9984 |
| Ferulic acid | 4.94 – 69.85 | 0.0203 ± 0.0005 | -0.0147 | 0.9980 |
| Caffeic acid | 16.48 – 233.08 | 0.0118 ± 0.0006 | -0.0298 | 0.9982 |
| Gallic acid | 9.69 – 137.05 | 0.0010 ± 0.00002 | 0.0012 | 0.9976 |
| Vanillic acid | 24.27 – 343.32 | 0.0111 ± 0.0006 | -0.0233 | 0.9973 |
| Para-coumaric acid | 2.63 – 37.26 | 0.0751 ± 0.0005 | -0.0272 | 0.9988 |
| Scopoletin | 8.16 – 115.49 | 2.4640 ± 0.1364 | 0.2385 | 0.9986 |

Table S8 Calibration ranges of analytes determined in serum and blood cell samples. The lowest data point of the respective calibration range corresponds to the validated LLoQ

| **Analyte** |  | **Calibration range** | | | |
| --- | --- | --- | --- | --- | --- |
|  | **Serum** | | |  | **Blood cells** |
| Caffeic acid | 1.0 – 52 ng/mL | | 5.5 – 289 nM |  | 16 – 233 ng/g |
| Coniferaldehyde | 0.6 – 69 ng/mL | | 3.4 – 387.2 nM |  | n.d. |
| Ferulic acid | 1.9 – 210 ng/mL | | 9.8 – 1081 nM |  | 5 – 70 ng/g |
| Gallic acid | 4.4 – 480 ng/mL | | 26 – 2822 nM |  | 10 – 137 ng/g |
| p-Coumaric acid | 1.0 – 105 ng/mL | | 6.1 – 639 nM |  | 3 – 37 ng/g |
| Protocatechuic acid | 13 – 1380 ng/mL | | 84 – 8955 nM |  | n.d. |
| Scopoletin | 6.3 – 173 ng/mL | | 33 – 900 nM |  | 8 – 115 ng/g |
| Sinapin aldehyde | 0.9 – 103 ng/mL | | 4.3 – 495 nM |  | n.d. |
| Syringaldehyde | 0.3 – 35 ng/mL | | 1.6 – 192 nM |  | n.d. |
| Syringic acid | 4.7 – 517 ng/mL | | 24 – 2608 nM |  | 15 – 207 ng/g |
| Urolithin A | 0.9 – 103 ng/mL | | 3.9 – 451 nM |  | 1 – 14 ng/g |
| Urolithin B | 0.5 – 53 ng/mL | | 2.4 – 250 nM |  | 0,6 – 9 ng/g |
| Urolithin C | 0.6 – 34 ng/mL | | 2.5 – 139 nM |  | 3 – 46 ng/g |
| Urolithin D | 0.9 – 100 ng/mL | | 3.5 – 384 nM |  | 5 – 66 ng/g |
| Vanillic acid | 17 – 944 ng/mL | | 101 – 5612 nM |  | 24 – 343 ng/g |
| Vanillin | 1.9 – 105 ng/mL | | 12 – 690 nM |  | n.d. |

n.d.: not determined

**Table S9.** Baseline serum concentrations in the Robuvit^®^ and placebo group. All other analytes revealed concentrations below the lower limit of quantification.

| Analyte | Robuvit^®^ | Placebo |
| --- | --- | --- |
| Caffeic acid | 1.17 [0.25] | 1.18 [0.19] |
| Ferulic acid | 4.46 [1.77] | 4.55 [1.59] |
| p-Coumaric acid | 0.98 [-] | < LLoQ |
| Protocatechuic acid | 80.8 [40.3] | 87.6 [44.7] |
| Scopoletin | 14.7 [3.32] | 14.2 [2.57] |
| Syringaldehyde | 0.49 [0.04] | < LLoQ |
| Syringic acid | 6.98 [1.48] | 7.14 [2.23] |
| Urolithin A | 1.80 [0.88] | 5.80 [12.8] |
| Urolithin B | 1.22 [0.47] | < LLoQ |
| Urolithin C | 1.69 [0.07] | 0.71 [-] |
| Vanillic acid | 33.4 [4.44] | 33.0 [5.23] |
| Vanillin | 3.42 [0.98] | 3.58 [0.90] |

Concentration of the analytes in serum (ng/mL) at baseline (t= 0). Numbers represent the median with interquartile range (IQR). No IQR is given when only one participant had detectable concentrations. LLoQ: Lower limit of quantification.

**Table S10.** Individual urolithin A serum concentrations (ng/mL) of patients at baseline, and after 4 and 8 weeks of intake of Robuvit^®^ or placebo, respectively.

| **Baseline** | |  | **4 weeks** | |  | **8 weeks** | |
| --- | --- | --- | --- | --- | --- | --- | --- |
| **Robuvit**^®^ | **Placebo** |  | **Robuvit**^®^ | **Placebo** |  | **Robuvit**^®^ | **Placebo** |
| 3.5 | 5.82 |  | 2.4 | 37.8 |  | 1.08 | 10.26 |
| 1.28 | 1.86 |  | 20.87 | 6.8 |  | 222.9 | 9.13 |
| 1.51 | 2.99 |  | 5.61 | 21.38 |  | 3.58 | 1.28 |
| 1.39 | 10.74 |  | 10.29 | 18.52 |  | 13.61 | 2.19 |
| 1.63 | 1.18 |  | 95.74 | 1.63 |  | 9.94 | 93.15 |
| 1.98 | 5.77 |  | 11.76 | 3.37 |  | 21.92 |  |
| 1.97 | 29.62 |  | 65.97 | 724.7 |  | 5.04 |  |
| 22.25 | 134.7 |  | 9.83 |  |  | 14.04 |  |
|  |  |  | 41.05 |  |  | 22.94 |  |
|  |  |  | 5.43 |  |  | 658.9 |  |
|  |  |  | 1.91 |  |  | 135.1 |  |
|  |  |  | 47.94 |  |  | 5.72 |  |
|  |  |  | 4.29 |  |  | 19.56 |  |
|  |  |  | 14.67 |  |  | 67.36 |  |
|  |  |  | 23.86 |  |  | 6.89 |  |
|  |  |  | 17.72 |  |  | 10.03 |  |
|  |  |  | 5.17 |  |  | 17.86 |  |
|  |  |  | 124.1 |  |  | 11.72 |  |
|  |  |  |  |  |  | 10.17 |  |
|  |  |  |  |  |  | 112.0 |  |
|  |  |  |  |  |  | 793.1 |  |

**Table S11.** Number of urolithin producers (Blood cells)

| Urolithin A |  |  |  |
| --- | --- | --- | --- |
|  | **t= 0** | **t= 4** | **t= 8** |
| Robuvit^®^ | **2** | **9** | **16** |
| Placebo | **2** | **4** | **3** |
| Urolithin B |  |  |  |
|  | **t= 0** | **t= 4** | **t= 8** |
| Robuvit^®^ | **1** | **4** | **6** |
| Placebo | **0** | **0** | **0** |

Number of patients in the Robuvit^®^ and placebo group with measurable urolithin A and B concentrations in blood cell samples from baseline (t= 0) until the end of the study (t= 8). Urolithin C was not detected in blood cells.
